# AIM‐Photonics Visible‐to‐Near‐Infrared Silicon‐Nitride‐Based Silicon‐Photonics Platform and Component Library

**DOI:** 10.1002/nap2.70350

**Published:** 2026-11-07

**Authors:** Andres Garcia Coleto, Henry Crawford‐Eng, Theodore J. Dahl, Mariel Shapiro, Maria Schnuck, Kyle J. Walsh, Steven T. Lipkowitz, Marcel W. Pruessner, Nathan F. Tyndall, Jacob N. Bouchard, Alin Antohe, Soumen Kar, Patrick J. O'Mullan, Cooper Hurley, Peter Hedlesky, Vijay S. S. Sundaram, Stefan Preble, Kevin McComber, James Eakin, Doug T. Petkie, Lewis G. Carpenter, Todd H. Stievater, Jelena Notaros

**Affiliations:** ^1^ Research Laboratory of Electronics Massachusetts Institute of Technology Cambridge Massachusetts USA; ^2^ Naval Research Laboratory Washington District of Columbia USA; ^3^ Amentum, 4800 Westfields Blvd, Suite 400 Chantilly Virginia USA; ^4^ American Institute for Manufacturing Integrated Photonics (AIM Photonics) Research Foundation SUNY Albany New York USA; ^5^ Department of Physics Worcester Polytechnic Institute Worcester Massachusetts USA; ^6^ Spark Photonics Design, Inc. Waltham Massachusetts USA; ^7^ Microsystems Engineering Rochester Institute of Technology Rochester New York USA

**Keywords:** integrated photonics, process design kit, silicon nitride, silicon photonics, visible integrated photonics

## Abstract

Silicon‐photonics platforms operating at visible and near‐infrared wavelengths are essential for a growing range of applications, including biosensing, chemical detection, Raman spectroscopy, quantum, optogenetics, and displays. However, unlike their infrared counterparts, visible‐wavelength platforms have lacked comprehensive component libraries with pre‐characterized devices, limiting the ability to rapidly design photonic integrated circuits for these applications. In this work, we present the design, fabrication, and broadband experimental characterization of a comprehensive visible‐to‐near‐infrared‐spanning component library for the AIM‐Photonics 300‐mm‐wafer silicon‐nitride‐based platform, which features two silicon‐nitride waveguiding layers, a sensing trench, thermo‐optic phase shifters for modulation, and a dicing trench for fiber‐to‐chip edge coupling. We design and characterize components for both the fundamental quasi‐transverse‐electric and quasi‐transverse‐magnetic modes for wavelengths spanning from 450 to 1000 nm. To enable this characterization, we utilize a spectrum‐spanning broadband measurement setup and methodology. The component library includes passive waveguides, edge couplers, grating couplers, multimode‐interferometer splitters, directional couplers with varying splitting ratios, waveguide crossings, interlayer‐transition escalators, polarization rotators, polarization splitters, sensing‐trench components, and thermo‐optic phase shifters. This work provides a foundry‐compatible pre‐characterized library of photonic building blocks that enables the rapid design and deployment of visible‐to‐near‐infrared photonic integrated circuits for sensing, quantum, biomedical, display, and other emerging applications.

## Graphical abstract

A comprehensive component library is presented for a 300‐mm‐wafer silicon‐nitride foundry platform operating at wavelengths spanning from 450 to 1000 nm. Waveguides, couplers, splitters, crossings, interlayer transitions, polarization devices, sensing‐trench structures, and thermo‐optic phase shifters are experimentally characterized across four spectral bands for both polarizations, providing pre‐characterized building blocks for visible light applications, such as sensing, quantum, biomedical, and display applications.

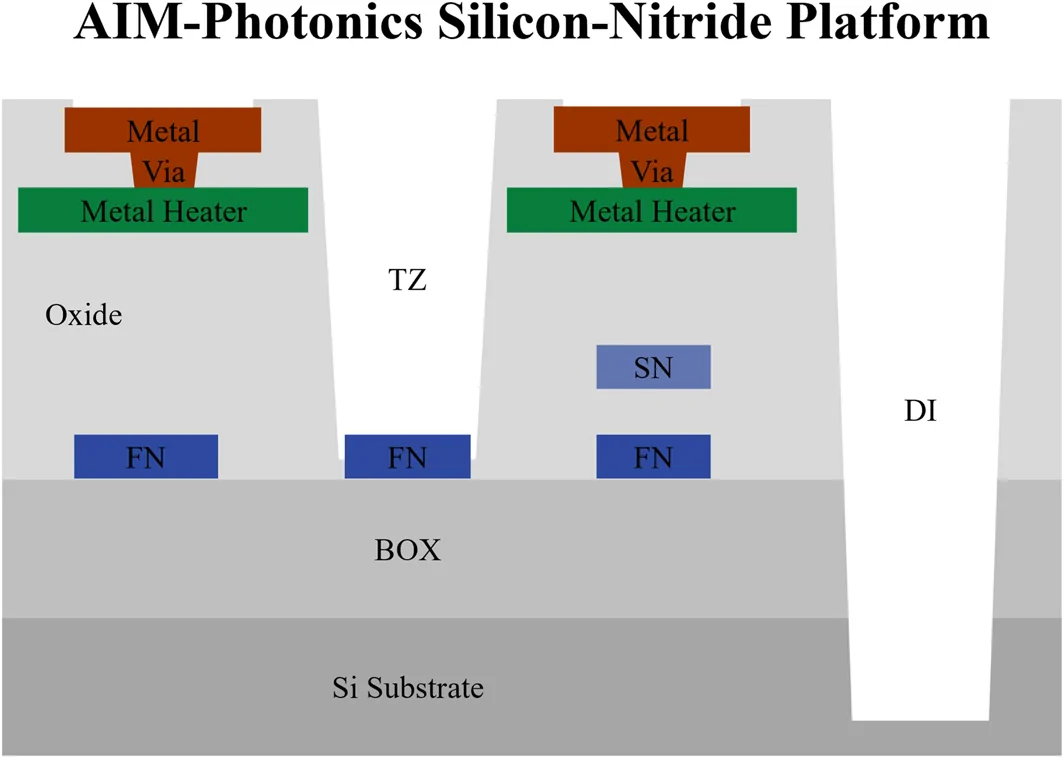

## 1 Introduction

The field of silicon photonics operating at infrared wavelengths has matured significantly over the past 2 decades, driven primarily by applications in optical communications [1, 2, 3] and light detection and ranging (LiDAR) [4, 5, 6, 7]. This progress has been enabled in large part by the development of high‐volume wafer‐scale fabrication platforms, particularly silicon‐on‐insulator platforms operating at telecom wavelengths, which have established robust foundry ecosystems with comprehensive process design kits (PDKs) and component libraries [8, 9, 10, 11]. These component libraries provide designers with a full suite of pre‐characterized photonic building blocks, including waveguides, couplers, splitters, modulators, and detectors, enabling rapid prototyping and scalable deployment of photonic integrated circuits for infrared applications. The availability of these mature PDKs and component libraries offered through multiproject wafer (MPW) services at foundries, such as the American Institute for Manufacturing Integrated Photonics (AIM Photonics) [8, 10, 11], has been instrumental in accelerating the adoption of silicon photonics in both academic research and commercial products.

More recently, there has been a growing shift toward silicon photonics operating at visible and near‐infrared wavelengths, driven by emerging application areas that require operation below the traditional telecom bands. These applications span a wide range of fields including biosensing [12, 13, 14], chemical sensing and waveguide‐enhanced Raman spectroscopy (WERS) [15, 16, 17, 18, 19], quantum information processing and sensing [20, 21, 22, 23], augmented‐ and virtual‐reality displays [24, 25, 26], and 3D printing [27]. Critically, unlike infrared‐wavelength silicon‐photonics applications, where most systems operate within a narrow spectral window around either 1310 nm or 1550 nm, visible‐wavelength applications span a broad spectral range with highly‐varying wavelength and bandwidth requirements. For example, trapped‐ion quantum systems require light at precisely‐defined atomic‐transition wavelengths, which can range from blue (such as 422 nm for strontium) to the near‐infrared (such as 650 nm for barium), demanding narrowband but wavelength‐specific component performance across a wide spectral range [20, 21, 22, 23]. In contrast, WERS requires a laser pump, sometimes preferring shorter pump wavelengths to maximize Raman scattering cross sections; however, the exact pump wavelength is less critical than the need for broadband low‐loss performance across the resulting Stokes‐shifted sensing fingerprint, which can span hundreds of nanometers [15, 16, 17, 18]. Augmented‐reality displays present yet another set of requirements, needing components that operate at blue, green, and red wavelengths simultaneously to produce RGB images, where the exact wavelengths are flexible, but performance across all 3 color bands is essential [24, 25, 26]. This diversity in both target wavelengths and bandwidth requirements means that a single operating wavelength cannot serve the wide range of applications that visible‐wavelength silicon photonics has the potential to impact. Instead, visible‐wavelength silicon‐photonics platforms must support broadband operation across hundreds of nanometers, with well‐characterized component performance spanning the full visible‐to‐near‐infrared spectrum. Despite this demand, visible‐wavelength silicon‐photonic platforms remain significantly underdeveloped compared to their infrared counterparts, and comprehensive component libraries with pre‐characterized devices spanning the visible spectrum are largely missing.

Silicon nitride (SiN) has emerged as one of the main materials of choice for visible‐wavelength silicon photonics due to its broadband optical transparency from the ultraviolet through the near‐infrared, low optical absorption across the visible spectrum, suitable refractive index for high‐confinement waveguiding, and compatibility with complementary metal‐oxide‐semiconductor (CMOS) fabrication processes [28]. Several silicon‐nitride‐based platforms have been demonstrated for visible‐wavelength operation [29, 30, 31, 32, 33]. However, many of these platforms have been fabricated using low‐volume custom processes, non‐CMOS‐compatible techniques, such as electron‐beam lithography, or smaller 200‐mm‐wafer processes that lack the throughput and small minimum feature sizes required for high‐volume manufacturing of visible‐wavelength silicon photonics. One key silicon‐photonics foundry in the United States is AIM Photonics, which operates a 300‐mm wafer fabrication facility with deep‐UV 193‐nm immersion lithography capable of sub‐100‐nm feature sizes [8, 10, 11]. Although our team has done some preliminary development of a silicon‐nitride platform at AIM Photonics with the capabilities necessary to support visible‐wavelength photonics, a comprehensive visible‐to‐near‐infrared‐compatible PDK and component library (analogous to the mature libraries available for infrared platforms) has been lacking.

In this paper, we present the design, fabrication, and broadband experimental characterization of a comprehensive visible‐to‐near‐infrared‐spanning component library for the AIM‐Photonics 300‐mm‐wafer silicon‐nitride‐based platform, which features two silicon‐nitride waveguiding layers, a sensing trench, thermo‐optic phase shifters for active modulation, and a dicing trench for smooth fiber‐to‐chip edge coupling. We design and characterize components for both the fundamental quasi‐transverse‐electric (TE) and quasi‐transverse‐magnetic (TM) modes for wavelengths spanning from 450 to 1000 nm and covering four spectral bands: violet/blue (V/B), green/yellow (G/Y), orange/red (O/R), and near‐infrared (I/Z). To enable this broadband characterization, we utilize a spectrum‐spanning measurement setup and methodology capable of testing devices across the full 450‐to‐1000‐nm wavelength range. The component library includes passive waveguides with propagation‐loss characterization across multiple waveguide widths (with TE single‐mode propagation losses as low as 4.5 dB/cm in the V/B band and decreasing to 0.1 dB/cm in the I/Z band), broadband fiber‐to‐chip edge couplers (with insertion losses as low as 4.3 dB/facet in the V/B band and decreasing to 1.9 dB/facet in the I/Z band), fiber‐to‐chip grating couplers (with insertion losses as low as 5.4 dB/coupler), 1‐x‐2 and 2‐x‐2 multimode‐interferometer (MMI) splitters, directional couplers with 50/50, 90/10, and 99/1 splitting ratios, low‐crosstalk waveguide crossings, interlayer‐transition escalators for adiabatic transitions between the two silicon‐nitride layers, polarization rotators, polarization splitters, sensing‐trench transitions and propagation structures, and thermo‐optic phase shifters. The measured performance of every component across the 4 bands is summarized in Table 1. This work provides the silicon‐photonics community with a foundry‐based pre‐characterized library of photonic building blocks for visible‐to‐near‐infrared applications, enabling the rapid design and deployment of visible‐to‐near‐infrared photonic integrated circuits for sensing, quantum, biomedical, display, and other emerging applications.

**TABLE 1:** Summary of the measured best performance of the component‐library devices for designs targeting each of the V/B, G/Y, O/R, and I/Z bands and both TE and TM operation.

Device and metric	V/B		G/Y		O/R		I/Z	
	TE	TM	TE	TM	TE	TM	TE	TM
Waveguide propagation loss (dB/cm)	4.5	3.1	1.6	0.9	0.7	0.15	0.1	0.1
Edge‐coupler insertion loss (dB/facet)	4.3	5.1	3.8	3.9	2.9	2.9	1.9	2.0
Grating‐coupler peak insertion loss (dB/coupler)	7.6	10.8	5.7	11.0	5.5	12.3	5.4	10.4
Grating‐coupler 3‐dB bandwidth (nm)	27	30	32	25	21	23	30	27
1‐x‐2 MMI‐splitter peak transmission (%)	98	93	97	96	97	99	> 99	> 99
2‐x‐2 MMI‐splitter splitting ratio (%)	50.1/49.9	50.1/49.9	50.0/50.0	49.7/50.3	49.9/50.1	51.0/49.0	50.0/50.0	49.9/50.1
50/50 directional‐coupler splitting ratio (%)	—	—	56.1/43.9	55.0/45.0	51.7/48.3	50.2/49.8	50.0/50.0	50.0/50.0
90/10 directional‐coupler splitting ratio (%)	—	—	93.5/6.5	90.6/9.4	92.0/8.0	90.0/10.0	90.0/10.0	90.0/10.0
99/1 directional‐coupler splitting ratio (%)	—	—	99.0/1.0	99.0/1.0	99.0/1.0	99.0/1.0	99.0/1.0	99.0/1.0
Waveguide‐crossing peak transmission (%)	98.6	98.5	98.9	98.6	99.1	98.4	99.6	99.3
Inter‐layer‐escalator peak transmission (%)	89.6	81.5	95.4	90.7	98.4	93.8	99.1	97.3
Polarization‐rotator conversion extinction (dB)	—	—	15.5	13.9	18.3	16.5	20.0	17.1
Polarization‐splitter extinction (dB)	—	—	18.2	16.6	18.8	20.4	21.1	22.0
Sensing‐trench transition loss (dB/transition)	—	—	0.25	0.67	0.37	1.27	0.43	—
Sensing‐trench propagation loss (dB/cm)	—	—	1.74	1.22	1.73	0.38	0.45	—
Thermo‐optic‐phase‐shifter P_π_ (mW)	43.7	46.4	51.3	57.7	67.2	80.7	89	112

*Note:* Each value is the weighted average across the measured reticles, evaluated at the best‐performing wavelength within the corresponding band. The waveguide propagation losses correspond to the single‐mode waveguide widths targeting each band (250‐nm‐wide for V/B, 300‐nm‐wide for G/Y, 400‐nm‐wide for O/R, and 800‐nm‐wide for I/Z). The grating‐coupler values are given for the optimal fiber angle within the measured 5°–15° range. The 1‐x‐2 MMI‐splitter transmissions are given for the best performing of the 3‐, 5‐, and 7‐mode designs. The 2‐x‐2 MMI‐splitter and direction‐coupler splitting ratios are given as thru‐port/cross‐port percentages. The polarization‐rotator conversion extinction values are given for TE‐to‐TM (TE column) and TM‐to‐TE (TM column) conversions. The polarization‐splitter extinction values are given for TE and TM inputs. The sensing‐trench values correspond to air‐clad measurements of 400‐nm‐wide, 500‐nm‐wide, and 800‐nm‐wide waveguides for the G/Y, O/R, and I/Z bands, respectively. The thermo‐optic‐phase‐shifter values correspond to the standard nonundercut heater designs with 250‐nm‐wide, 300‐nm‐wide, 400‐nm‐wide, and 800‐nm‐wide waveguides for the V/B, G/Y, O/R, and I/Z bands, respectively.

## 2 Platform and Fabrication

In this section, we describe the AIM‐Photonics 300‐mm‐wafer silicon‐nitride‐based platform, fabricated by AIM Photonics within the Albany NanoTech Complex (ANC). All wafer‐level processing is performed using a suite of advanced 300‐mm‐wafer processes for 193‐nm immersion lithography, thin‐film chemical vapor deposition (CVD), chemical‐mechanical polishing (CMP), and reactive‐ion etch (RIE). The platform consists of two vertically‐separated silicon‐nitride (SiN) waveguiding layers clad in silicon dioxide (SiO_2_), a sensing trench, heaters to enable active thermo‐optic phase shifters, and a dicing trench for smooth fiber‐to‐chip edge coupling, all fabricated on a 300‐mm‐diameter silicon wafer substrate as shown in Figure 1a.

**FIGURE 1:**
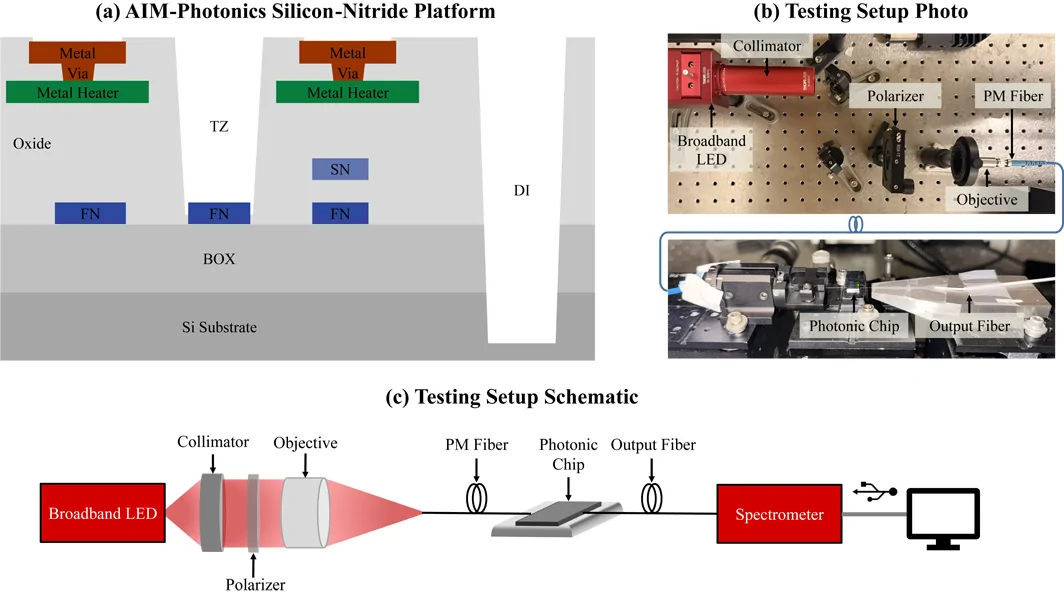
(a) Simplified cross‐sectional schematic of the AIM‐Photonics silicon‐nitride platform consisting of two silicon‐nitride layers (FN and SN), a metal heater, a sensing trench (TZ), and a dicing trench (DI) (not to scale). (b) Photograph and (c) schematic of the broadband testing setup with the relevant components labeled.

First, a high‐purity buried‐oxide (BOX) layer is grown on top of the silicon substrate to provide optical isolation from the silicon handle wafer. On top of this buried oxide, the first silicon‐nitride waveguiding layer, referred to as the first nitride (FN), is deposited using a CVD process and planarized. The FN layer is then patterned using 193‐nm immersion lithography and a dry‐etch process to define the device geometries. A silicon‐dioxide layer is then deposited to encapsulate the FN layer, and its top surface is planarized using a CMP step. The second silicon‐nitride waveguiding layer, referred to as the second nitride (SN), is subsequently deposited using a CVD process and planarized. The SN layer is then patterned analogously to the FN layer. A high‐temperature annealing step is used to reduce both loss and fluorescence of the silicon‐nitride waveguiding layers [34, 35]. Next, a thick silicon‐dioxide top cladding layer is deposited, and its top surface is planarized using a CMP step, encapsulating both waveguiding layers in silicon dioxide. The resulting silicon‐nitride waveguiding layers have a refractive index of approximately 2.03 at 450 nm, 2.01 at 520 nm, 2.00 at 633 nm, and 1.98 at 785 nm, providing high index contrast with the surrounding silicon dioxide for high‐confinement waveguiding across the visible‐to‐near‐infrared spectrum.

Above the silicon‐dioxide cladding, a metallization stack is fabricated to enable active thermo‐optic devices using a series of additional etch‐stop layers, trenches, metal depositions, and silicon‐dioxide depositions. First, a resistive metal layer is deposited and patterned to define resistive heater elements above the waveguides. A metal via layer is then formed to provide electrical contact from the heaters up to the top metal layer. A metal layer is then fabricated to define electrical routing traces and top‐level electrical pads for interfacing with electrical probes or wire bonding. This metallization stack enables thermo‐optic phase shifting by resistively heating the waveguide region, locally modifying the refractive index of the waveguide materials through the thermo‐optic effect [36, 37].

To enable sensing applications that require direct interaction between the guided optical mode and an external medium, a sensing trench (TZ) is included in the platform. Specifically, the top silicon‐dioxide cladding is etched down to the FN layer, exposing the FN layer to the environment above the chip. This allows the evanescent field of the guided mode in the FN layer to interact directly with an analyte, which is critical for applications such as WERS and biosensing, where the strength of the light–matter interaction determines the sensitivity of the sensor [15, 16, 17, 18, 19].

To enable fiber‐to‐chip coupling, a deep dicing trench (DI) is etched into the silicon‐dioxide cladding at the edges of each chip, extending down into the silicon substrate. This dicing trench defines the chip boundary and creates a smooth facet for low‐loss fiber‐to‐chip edge coupling after dicing, eliminating the need for postfabrication polishing, and enabling high‐quality optical facets directly from the wafer‐scale fabrication process.

Finally, the silicon‐dioxide above each electrical pad is removed to create an opening for each pad, and the wafer is diced along the DI trenches to separate the individual chips for testing or packaging.

Note that the vertical dimensions of the platform (including the silicon‐nitride layer thicknesses, the inter‐layer dielectric spacing, and the top cladding thickness) are proprietary to AIM Photonics and, therefore, are not disclosed in this publication. This information is available to authorized platform users through the AIM Photonics PDK. Additional platform details, including layer‐thickness values reported for related versions of this silicon‐nitride process, can be found in refs. [11, 34, 38].

## 3 Characterization Methodology

To characterize the component library across the full 450‐to‐1000‐nm spectral range, we utilize a spectrum‐spanning broadband characterization technique and measurement setup [39]. Unlike infrared silicon photonics, where devices are typically characterized at a single wavelength or within a narrow spectral window, such as the telecom C‐band (1530–1565 nm) or O‐band (1260–1360 nm), visible applications can span hundreds of nanometers and target highly diverse wavelengths. Therefore, characterizing a visible‐wavelength component library at a single wavelength is insufficient; instead, broadband characterization across the entire operating range is essential to capture the wavelength‐dependent performance of each device and to provide designers with comprehensive spectral data for system‐level design.

To perform this broadband characterization, we use a custom‐built measurement setup as shown in Figure 1b,c. This setup employs two broadband sources to span the full spectral range of the component library: a Thorlabs SLS201L broadband light source for the I/Z band (700–1000 nm) and a Thorlabs MCWHLP2 broadband white‐light LED for the V/B, G/Y, and O/R bands (450–700 nm). The broadband source output is collimated and passed through a linear polarizer to ensure high polarization purity. The output light is then coupled into a polarization‐maintaining (PM) fiber to establish a well‐defined input polarization state for each measurement. We use a Thorlabs PM‐S405‐XP PM fiber for the V/B and G/Y bands (with a 3.3‐to‐4.6‐μm mode‐field diameter across its 400‐to‐680‐nm operating range), a Thorlabs PM630‐HP PM fiber for the O/R band (with a 4.5‐μm mode‐field diameter at 630 nm), and a Thorlabs PM780‐HP PM fiber for the I/Z band (with a 5.3‐μm mode‐field diameter at 780 nm), selected to maintain single‐mode polarization‐maintaining operation within their respective wavelength ranges.

To assist in aligning the input beam to the chip edge couplers, we have the option to use a red laser, such as a He–Ne laser. This alignment laser provides a visible bright spot on the chip facet, enabling precise positioning of the input beam onto the target waveguide edge coupler. Once alignment is established, the setup can be toggled to route either broadband source into the input fiber for the characterization measurements.

At the output of the chip, the light exiting the output edge coupler is collected and coupled into a single‐mode output fiber (matching the input fiber). This output fiber is connected to a Wasatch Photonics WP‐VISNIR‐C‐IC spectrometer which records the full output spectrum in a single acquisition across the visible‐to‐near‐infrared range. By recording the complete broadband transmission spectrum through each device under test, we obtain the wavelength‐dependent insertion loss, splitting ratio, or other relevant performance metrics across the full operating bandwidth of the device in a single measurement. By rotating the input polarization and repeating the measurement, we independently characterize the performance for both the TE and TM input modes.

This broadband characterization procedure is repeated for each device across multiple chips, reticles, and wafers to enable statistical analysis of the component performance and to ensure capture of any fabrication‐induced variability in the platform.

## 4 Waveguide Routing: Waveguide Propagation and Bends

We first characterize the propagation loss of waveguides on the FN layer, which serves as the primary waveguiding layer for the component library. Propagation loss is a key metric for any silicon‐photonics platform as it directly impacts the performance of many devices and sets limits on system‐level loss budgets.

To measure the propagation loss, we use a set of paperclip test structures consisting of four individual waveguides of varying lengths, analogous to those described in our prior work [40]. The paperclip test structures are designed such that the total waveguide propagation length changes, but the number of loop‐back curves remains constant across all four structures, negating the effect of waveguide bend loss in the measurement. For each waveguide width and polarization, we measure the broadband transmitted optical power spectrum through all four paperclip structures using the characterization setup described in Section 3. At each wavelength, we then perform a linear fit of the measured transmitted power (in dB) versus waveguide length, with the slope of this fit directly yielding the propagation loss in dB/cm. This procedure is repeated across three reticles on the wafer, and the results are combined to obtain a weighted average and standard deviation of the propagation loss at each wavelength, capturing both the spectral performance dependence of the waveguides and the fabrication‐induced variability across the wafer. Note that, for wavelengths below approximately 500 nm, the fiber‐to‐chip coupling procedure described in Section 3 is too lossy to provide an adequate signal‐to‐noise ratio, so we instead couple the input light onto the chip using free‐space coupling; then, we stitch together the free‐space and fiber‐coupled data near 500 nm, which can produce small discontinuities in the extracted mean and standard deviation at this stitching boundary.

We characterize five different waveguide widths in the FN layer for both TE and TM input polarizations: 250 nm targeting the V/B band, 300 nm targeting the G/Y band, 400 and 500 nm targeting the O/R band, and 800 nm targeting the I/Z band, each selected to maintain single‐mode operation within the majority of its respective target spectral band. The measured propagation losses as a function of wavelength for all 5 waveguide widths and both polarizations are shown in Figure 2, with the solid lines representing the weighted averages across 3 reticles and the shaded regions representing the 1‐σ standard deviations.

**FIGURE 2:**
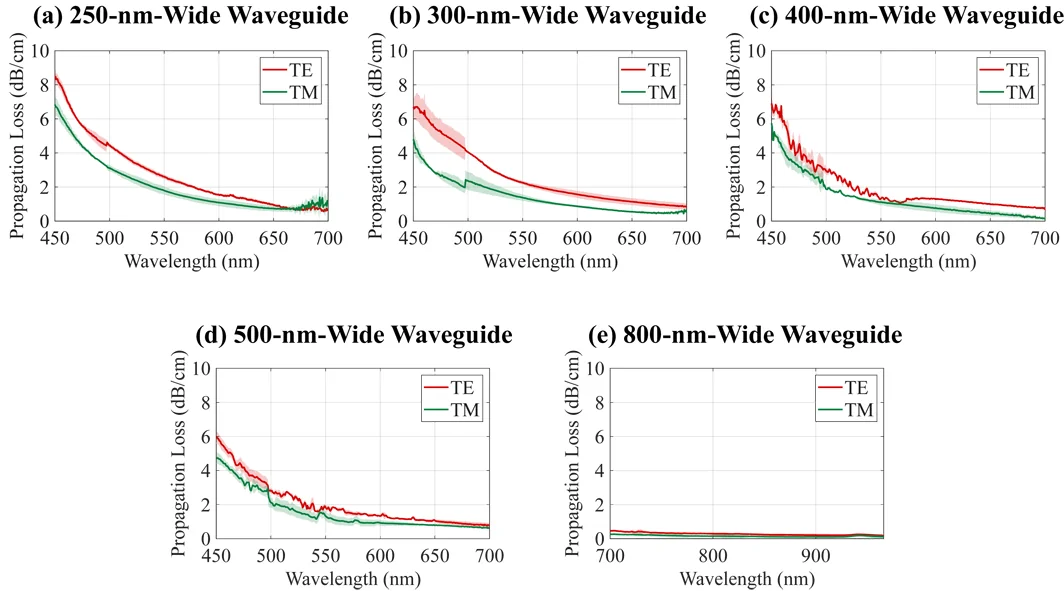
Measured waveguide propagation loss (dB/cm) as a function of wavelength in the FN layer for (a) 250‐nm‐wide, (b) 300‐nm‐wide, (c) 400‐nm‐wide, (d) 500‐nm‐wide, and (e) 800‐nm‐wide waveguides for both TE (red) and TM (green) operation. Solid lines represent the weighted average across 3 reticles, and shaded regions represent the 1‐σ standard deviation.

As expected, the propagation loss generally increases at shorter wavelengths for all waveguide widths. Two primary loss mechanisms contribute to this measured propagation loss: scattering loss due to sidewall roughness and material absorption loss. The scattering loss is caused primarily by line‐edge roughness on the waveguide sidewalls introduced during the lithographic patterning and etching processes. The wavelength dependence of this scattering loss is governed by the intrinsic Rayleigh‐scattering scaling (proportional to *λ* ^−4^) and the wavelength‐dependent overlap of the guided mode with the sidewall roughness, resulting in generally stronger scattering at shorter wavelengths for a given roughness level. This scattering loss is also more pronounced for narrower waveguides, where the optical mode interacts more strongly with the sidewalls.

Importantly, unlike other low‐loss silicon‐nitride platforms that achieve low propagation losses through weakly‐confined ultrathin cores, which require millimeter‐scale bend radii due to the weak mode confinement [41], the FN layer in this platform provides sufficient mode confinement to support tight bend radii typically below 100 μm across the operating wavelength range. For example, in the worst‐case scenario (TM polarization at the longest operating wavelength in each band), the minimum bend radius for less than 1% loss per 90° bend is 25 μm for the 250‐nm‐wide waveguide at 500 nm, 35 μm for the 300‐nm‐wide waveguide at 600 nm, 65 μm for the 400‐nm‐wide waveguide at 750 nm, and 95 μm for the 800‐nm‐wide waveguide at 900 nm. This is a critical advantage for the development of a practical component library as compact bend radii enable dense device layouts and small chip footprints, which are essential for complex photonic integrated circuits.

Additionally, this platform exhibits low background fluorescence, which has been characterized in detail in our prior work [34, 35]. Low background fluorescence is critical for applications in waveguide‐enhanced Raman spectroscopy and quantum sensing, where weak optical signals from analytes or single‐photon emitters must be detected above the intrinsic background emission of the waveguide. The combination of low propagation loss, compact bend radii, and low background fluorescence makes this platform well‐suited for high‐sensitivity sensing and quantum applications across the visible‐to‐near‐infrared spectrum.

## 5 Fiber‐to‐Chip Coupling: Edge and Grating Couplers

Efficient coupling of light between optical fibers and the photonic chip is essential for all measurements and applications of the component library. We design and characterize two types of fiber‐to‐chip couplers: broadband edge couplers for high performance across the full spectral range and grating couplers for wafer‐scale testing where dicing and facet access are not feasible.

### 5.1 Edge Couplers

Edge couplers are the preferred fiber‐to‐chip coupling solution for this component library due to their broadband operation and polarization insensitivity. Unlike grating couplers, which rely on diffraction and are inherently narrowband and polarization dependent, edge couplers can support both the TE and TM modes across an entire spectral band with low loss, making them well suited for the broadband characterization methodology described in Section 3.

The edge couplers in this library are based on an inverse‐taper design, in which the waveguide width is adiabatically tapered down to a narrow tip at the chip facet [42]. The narrow tip expands the guided optical mode to better match the mode‐field diameter of the input fiber, improving the overlap between the fiber mode and the waveguide mode and, therefore, reducing the coupling loss. We design one edge coupler to target each spectral band, with the taper geometry optimized at the target wavelength of that band. For example, narrower tip widths are used for the designs targeting the shorter‐wavelength bands (e.g., 70‐nm tip width for the V/B edge coupler), where the fiber mode‐field diameters are smaller and tighter mode confinement is required, whereas wider tips are used for the designs targeting the longer‐wavelength bands (e.g., 140‐nm tip width for the I/Z edge coupler). Because the polarization dependence of an inverse‐taper design is weak, a single design serves both the TE and TM modes within each band. The inverse taper then transitions adiabatically from the narrow tip to the standard single‐mode waveguide width for the corresponding band over a 200‐μm length, with the length chosen to ensure low transition loss. The edge‐coupler parameters for designs targeting each of the V/B, G/Y, O/R, and I/Z bands are summarized in Table 2.

**TABLE 2:** Parameters for the inverse‐taper edge couplers for designs targeting each of the V/B, G/Y, O/R, and I/Z bands (a single design serves both the TE and TM polarizations within each band).

Parameter	V/B	G/Y	O/R	I/Z
Tip width	70 nm	90 nm	110 nm	140 nm
Taper length	200 µm	200 µm	200 µm	200 µm
Waveguide width	250 nm	300 nm	400 nm	800 nm

The edge couplers are accessed through the dicing trench described in Section 2. The trench provides a smooth and well‐controlled facet interface at the edge‐coupler termination, enabling low‐loss edge coupling directly from the wafer‐scale process without the need for postfabrication polishing.

To characterize the edge‐coupler insertion loss, we measure the broadband transmitted power through a waveguide test structure consisting of an input edge coupler, a short straight waveguide with an S‐bend offset to avoid coupling scattered light at the output, and a second output edge coupler. We use the setup described in Section 3, and we use PM fibers that match the operating wavelengths: a Thorlabs PM‐S405‐XP PM fiber for the V/B and G/Y bands, a Thorlabs PM630‐HP PM fiber for the O/R band, and a Thorlabs PM780‐HP PM fiber for the I/Z band (with mode‐field diameters provided in Section 3). Next, we normalize the measured transmitted power through the test structure to a reference measurement obtained by bypassing the chip and connecting the input and output PM fibers directly, which removes the wavelength‐dependent response of the source, fibers, and spectrometer from the measurement. This yields the total insertion loss of the two edge couplers and the connecting waveguide. We then subtract the known propagation loss of the connecting waveguide (obtained from measurements in Section 4) and divide by two to obtain the per‐facet edge‐coupler insertion loss. We perform this measurement for both the TE and TM modes by adjusting the input fiber polarization orientation.

The measured insertion losses of the edge couplers as a function of wavelength for both the TE and TM input modes for the designs targeting each of the V/B, G/Y, O/R, and I/Z bands are shown in Figure 3, with the solid lines representing the weighted averages across 3 reticles and the shaded regions representing the 1‐σ standard deviations. Across all 4 bands, the coupling loss generally decreases at longer wavelengths, consistent with improved mode‐field overlap between the fiber mode and the expanded waveguide mode at the inverse taper tip as the wavelength increases. Additionally, the TE and TM modes exhibit comparable coupling losses across all bands, confirming the polarization insensitivity of the inverse‐taper edge couplers. The higher coupling losses and increased variability at shorter wavelengths are expected as the smaller fiber mode‐field diameters and tighter mode confinement at these wavelengths make the mode overlap more sensitive to the taper tip geometry and fabrication tolerances. The narrow standard‐deviation regions across most of the operating range, particularly in the O/R and I/Z bands, indicate good fabrication uniformity of the edge couplers across the wafer. In the future, the coupling losses at shorter wavelengths in the V/B and G/Y bands could be further reduced by optimizing the taper tip width or by using fibers with smaller mode‐field diameters that better match the edge‐coupler mode at these wavelengths.

**FIGURE 3:**
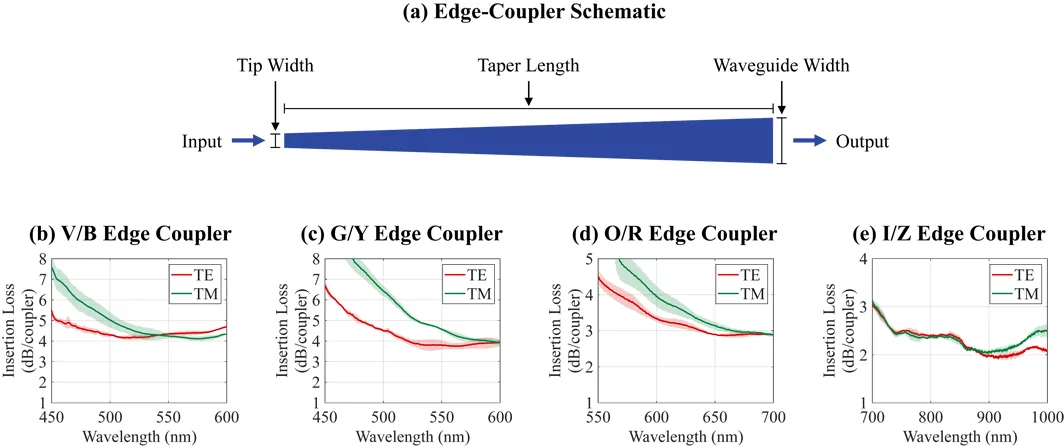
(a) Schematic of the inverse‐taper edge coupler, with key design parameters labeled. Measured insertion loss (dB/coupler) of the edge couplers as a function of wavelength for designs targeting the (b) V/B, (c) G/Y, (d) O/R, and (e) I/Z bands and both TE (red) and TM (green) operation. Solid lines represent the weighted average across 3 reticles, and shaded regions represent the 1‐σ standard deviation.

### 5.2 Grating Couplers

Grating couplers provide a complementary fiber‐to‐chip coupling solution that is useful for wafer‐scale testing and rapid prototyping, where dicing the wafer and accessing edge facets is not feasible. By coupling light vertically through the top surface of the chip, grating couplers enable optical testing of devices on intact wafers, facilitating high‐throughput characterization and screening the devices prior to dicing. However, because grating couplers rely on a periodic diffractive structure to couple light between the fiber and the waveguide, they are inherently narrowband and polarization dependent, with their peak coupling wavelength and efficiency determined by the grating period, duty cycle, and the angle of the fiber [43, 44, 45]. Therefore, the grating couplers in this library are designed to target specific laser lines commonly used in visible‐wavelength applications, such as 785 nm, 633 nm, 520 nm, and 450 nm, rather than providing broadband coupling across an entire spectral band.

The grating couplers are designed in the FN layer with curved grating teeth. A single‐mode input waveguide feeds into a taper that expands the mode laterally before entering the grating region. The curvature of the grating teeth is designed to match the lateral beam expansion from the taper, ensuring the diffracted wavefront remains phase matched across the full width of the grating and focuses into the fiber mode. The overall width of the grating region is optimized to match the mode‐field diameter of the target fiber, maximizing the spatial overlap between the diffracted field and the fiber mode. Then, the grating period and duty cycle are optimized using a particle swarm optimizer for each target wavelength and polarization to maximize the coupling efficiency at the design wavelength and for the desired fiber angle. We design separate grating couplers for both TE and TM mode operation for each of the 4 wavelength bands as the different polarizations require different grating parameters to achieve efficient diffraction into the fiber. The grating‐coupler parameters for designs targeting each of the V/B, G/Y, O/R, and I/Z bands and both TE and TM operation are summarized in Table 3.

**TABLE 3:** Parameters for the grating‐coupler designs targeting each of the V/B, G/Y, O/R, and I/Z bands and both TE and TM operation. The duty cycle is defined as the unetched fraction of the grating period.

Parameter	V/B		G/Y		O/R		I/Z	
	TE	TM	TE	TM	TE	TM	TE	TM
Design wavelength	450 nm		520 nm		633 nm		785 nm	
Grating period	324 nm	319 nm	368 nm	391 nm	455 nm	469 nm	577 nm	569 nm
Duty cycle	45%	51%	50%	47%	51%	60%	48%	62%
Grating‐coupler width	4.0 µm		5.0 µm		5.5 µm		5.5 µm	
Number of grating periods	40		40		40		40	
Waveguide width	250 nm		300 nm		400 nm		800 nm	
Fiber angle	10°		10°		10°		10°	

The testing setup for the grating couplers differs slightly from the setup described in Section 3. First, we replace the polarization‐maintaining fibers with standard single‐mode fibers and use polarization paddles to control the input polarization state. This approach provides higher polarization extinction ratios at the grating‐coupler input, since controlling the PM‐fiber rotational alignment in the angled fiber mounts (necessary for grating‐coupler measurements as described below) is more challenging than in the fixed edge‐coupling configuration described in Section 3. The fiber types are selected to provide single‐mode operation at each target wavelength: a Thorlabs 460HP fiber for both the V/B and G/Y bands (with a 3.5‐μm mode‐field diameter at 515 nm), a Thorlabs 630HP fiber for the O/R band (with a 4.0‐μm mode‐field diameter at 630 nm), and a Thorlabs 780HP fiber for the I/Z band (with a 5.0‐μm mode‐field diameter at 850 nm). We position the input fiber above the grating coupler at an angle using a custom angled fiber mount and a Thorlabs GNL18 goniometer. To measure the grating‐coupler insertion loss, we couple light from the input fiber through the input grating coupler into an on‐chip waveguide and collect the output through a second output grating coupler into an output fiber connected to the spectrometer. We vary both the input and output fiber angles (matching the output fiber angle to the input fiber angle) from 5° to 15° (with 0° being defined as perpendicular to the chip) and record the output power as a function of wavelength and fiber angle. Then, similarly to the edge‐coupler characterization described above, we normalize the measured transmitted power to the power we measure when bypassing the chip with a matching single‐mode fiber, and we extract the total insertion loss of the two grating couplers and divide by two to obtain the insertion loss per grating coupler.

The measured insertion losses of the grating couplers as a function of wavelength and fiber angle for designs targeting each of the V/B, G/Y, O/R, and I/Z bands and both TE and TM operation are shown in Figure 4, where the insertion loss values represent the average across four total measurements: two repeated measurements per test structure per design on each of two different reticles. Several key observations can be drawn from these results. First, the TE grating couplers consistently exhibit significantly better performance than the TM grating couplers across all four bands. This is because the TE modes have higher confinement in the grating regions, resulting in stronger scattering from the grating teeth, which enables better matching of the radiated field profiles to the small mode‐field diameters of the fibers. Second, for each band, the optimal coupling wavelength follows a clear and well‐defined trend with the fiber angle. As the fiber angle increases, the peak coupling wavelength shifts as expected from grating‐coupler theory [43, 44, 45]. This angular tunability can be a practical advantage as it allows users to adjust the fiber angle to shift the peak coupling to a specific wavelength of interest within the band, providing flexibility for applications targeting different laser lines. Third, we observe periodic fringes in the coupling efficiency as a function of wavelength, caused by Fabry–Perot interference between light coupled directly upward into the fiber and light that is first diffracted downward toward the substrate and then reflected back from the substrate‐BOX interface. This fringe modulation could be mitigated in the future using a dual‐layer grating‐coupler design with unidirectional emission to suppress the downward‐diffracted component [45, 46].

**FIGURE 4:**
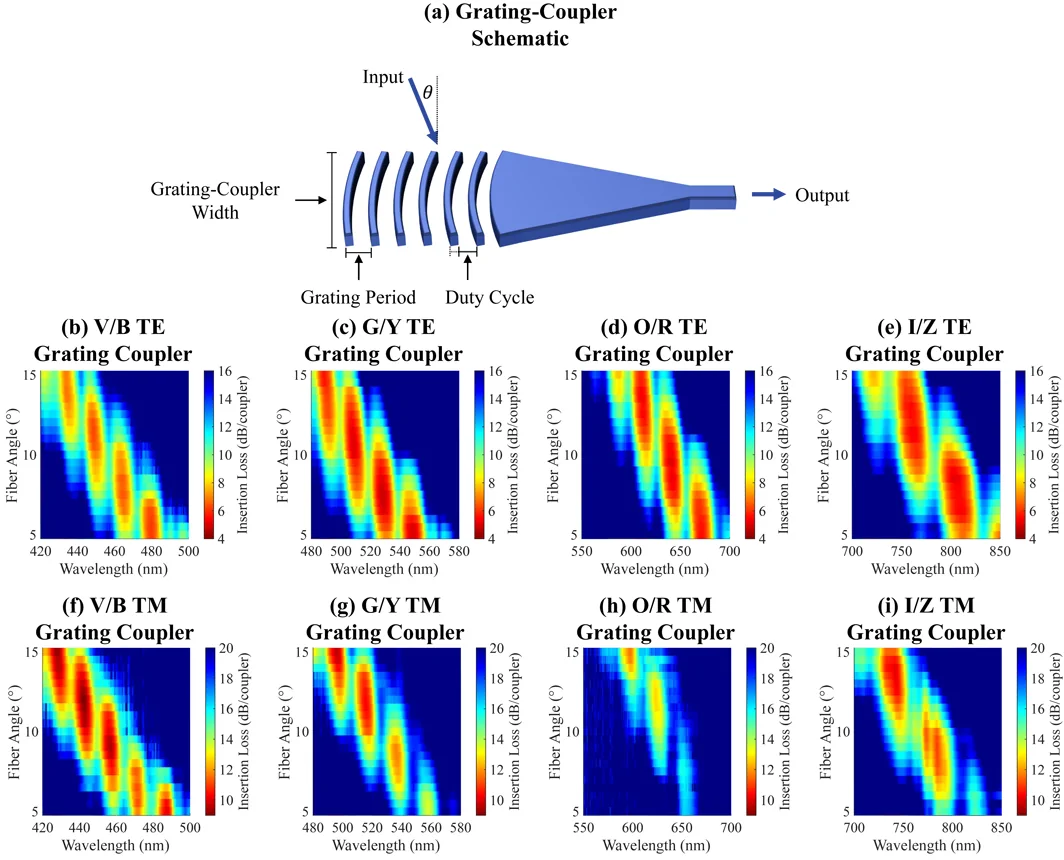
(a) Schematic of the grating coupler, with key design parameters labeled. Measured insertion loss (dB/coupler) of the grating couplers as a function of wavelength and fiber angle for designs targeting the V/B, G/Y, O/R, and I/Z bands and both (b–e) TE and (f–i) TM operation. Note the different colorbar scale for the TE versus TM measurements.

## 6 Power Splitting and Interference: MMI Splitters and Directional Couplers

Power splitting and combining devices are fundamental building blocks of any photonic component library, enabling signal distribution, interferometric systems, and power monitoring. We design and characterize three types of splitting devices: 1‐x‐2 multimode‐interferometer (MMI) splitters for even 50/50 power splitting, 2‐x‐2 MMI splitters for general 3‐dB coupling and mixing, and directional couplers with varying splitting ratios for flexible splitting and combining operations.

### 6.1 1‐x‐2 MMI Splitters

The 1‐x‐2 MMI splitters are designed to evenly split an input optical signal into two output ports with a 50/50 power ratio. This device is critical for signal distribution in photonic integrated circuits and serves as a fundamental component in interferometric devices such as Mach–Zehnder interferometers (MZIs). The 1‐x‐2 MMIs consist of a single input waveguide that feeds into a wider multimode region, where interference between the multiple excited modes produces two symmetric images of the input field at a specific propagation length, which are then captured by two output waveguides [47].

A key design tradeoff for the MMI splitter is the width of the multimode region. A wider multimode region supports more guided modes, which produces a sharper imaging of the modes, and, consequently, lower insertion loss at the design wavelength [47]. However, a wider multimode region also increases the device footprint, can introduce higher loss if the input and output waveguide positions are not precisely optimized, and will have a narrower operating bandwidth and higher sensitivity to fabrication variations. In contrast, a narrower multimode region supports fewer modes, resulting in a more compact device, larger operating bandwidth, and lower sensitivity to fabrication variations, but at the cost of an increase in the insertion loss since the self‐imaging point is not as sharp. To explore this tradeoff and provide users of the component library with the flexibility to select devices best suited to their application (whether prioritizing peak performance at a specific wavelength or broader operating bandwidth with relaxed fabrication sensitivity), we design 1‐x‐2 MMI splitters with three different multimode‐region widths, optimized to support 3, 5, and 7 guided modes at the target wavelength for each spectral band. Because the TE and TM modes have different effective indices and mode profiles within the multimode region, the optimal MMI length and waveguide positions differ for each polarization. Therefore, for each multimode‐region width and spectral band, we independently optimize two separate device designs, one for TE operation and one for TM. For each device, we optimize the length of the multimode region, the positions of the input and output waveguides, and the taper geometries connecting the single‐mode waveguides to the multimode region to maximize the total transmitted power at the design wavelength. The 1‐x‐2 MMI parameters for designs targeting each of the V/B, G/Y, O/R, and I/Z bands and both TE and TM operation are summarized in Table 4.

**TABLE 4:** Parameters for the 1‐x‐2 MMI splitter designs targeting each of the V/B, G/Y, O/R, and I/Z bands and both TE and TM operation. The output‐port gap is the edge‐to‐edge separation between the two output tapers.

Parameter	V/B		G/Y		O/R		I/Z	
	TE	TM	TE	TM	TE	TM	TE	TM
Multimode‐region width (3‐mode)	0.75 µm	0.90 µm	1.00 µm	1.25 µm	1.40 µm	1.75 µm	2.00 µm	2.75 µm
Multimode‐region width (5‐mode)	1.10 µm	1.40 µm	1.50 µm	1.85 µm	2.00 µm	2.65 µm	3.00 µm	4.00 µm
Multimode‐region width (7‐mode)	1.60 µm	1.85 µm	2.00 µm	2.45 µm	2.75 µm	3.55 µm	4.00 µm	5.00 µm
Multimode‐region length (3‐mode)	1.349 µm	2.046 µm	2.047 µm	3.204 µm	3.152 µm	5.079 µm	5.015 µm	9.381 µm
Multimode‐region length (5‐mode)	2.690 µm	4.392 µm	4.218 µm	6.459 µm	5.920 µm	10.507 µm	10.493 µm	18.870 µm
Multimode‐region length (7‐mode)	5.391 µm	7.339 µm	7.148 µm	10.760 µm	10.839 µm	18.006 µm	18.002 µm	28.416 µm
Output‐port gap (3‐mode)	0.103 µm	0.166 µm	0.147 µm	0.267 µm	0.241 µm	0.406 µm	0.134 µm	0.114 µm
Output‐port gap (5‐mode)	0.197 µm	0.334 µm	0.305 µm	0.464 µm	0.426 µm	0.714 µm	0.315 µm	0.579 µm
Output‐port gap (7‐mode)	0.384 µm	0.497 µm	0.495 µm	0.694 µm	0.709 µm	1.062 µm	0.298 µm	0.767 µm
Taper width (3‐mode)	0.313 µm	0.349 µm	0.403 µm	0.449 µm	0.533 µm	0.598 µm	0.978 µm	0.998 µm
Taper width (5‐mode)	0.383 µm	0.432 µm	0.485 µm	0.542 µm	0.628 µm	0.730 µm	1.277 µm	1.595 µm
Taper width (7‐mode)	0.456 µm	0.494 µm	0.555 µm	0.621 µm	0.729 µm	0.842 µm	1.794 µm	1.933 µm
Taper length	10 µm		10 µm		10 µm		25 µm	

To characterize the 1‐x‐2 MMI splitters, we use cascaded splitter‐tree test structures. In these test structures, a single input waveguide feeds into a 1‐x‐2 MMI splitter. One output arm of this splitter is routed directly to an output edge coupler, whereas the other output arm feeds into the next 1‐x‐2 MMI splitter in the cascade. This process is repeated across four stages, producing a series of output edge couplers, each one stage deeper in the cascade. For an ideal lossless 50/50 splitter, each stage would reduce the power at the measured output by exactly 50%; however, in practice, each stage introduces additional loss beyond this ideal 50% splitting, such that any additional power reduction beyond this ideal 50% value corresponds to the insertion loss from the splitter. We measure the transmitted output power at each output edge coupler using the broadband characterization setup described in Section 3, and perform a linear fit of the measured power (in dB) versus the number of splitting stages to extract the insertion loss per MMI splitter. We then convert this extracted insertion loss from dB to linear scale, and multiply by two to account for the power in both output arms, such that an ideal lossless splitter yields a normalized transmission of 1. For each 1‐x‐2 MMI splitter design, we measure one 4‐stage cascade splitter test structure as described above on three reticles on the wafer.

The measured total normalized transmissions of the 1‐x‐2 MMI splitters as a function of wavelength for designs targeting each of the V/B, G/Y, O/R, and I/Z bands and both TE and TM operation are shown in Figure 5, with the solid lines representing the weighted averages across three reticles and the shaded regions representing the 1‐σ standard deviations. Several trends are observed in the measured data that are consistent with the expected design tradeoffs. Across all 4 bands and both polarizations, the widest 7‐mode MMIs generally achieve the highest peak transmission near their target design wavelength, but exhibit narrower band operation, with the transmission rolling off more steeply away from the design wavelength. The narrower 3‐mode MMIs, in contrast, maintain more uniform transmission over a broader wavelength range, but with a lower peak value, reflecting the less‐sharp self‐imaging produced by fewer guided modes. The 5‐mode MMIs provide an intermediate balance between peak performance and bandwidth. The TE and TM results show broadly similar trends, though there are some band‐specific differences in the relative performance of the three widths. The standard‐deviation regions are generally narrow across most bands, indicating good fabrication repeatability, with somewhat larger variability observed at shorter wavelengths and for the designs with wider multimode regions where fabrication sensitivity is expected to be higher.

**FIGURE 5:**
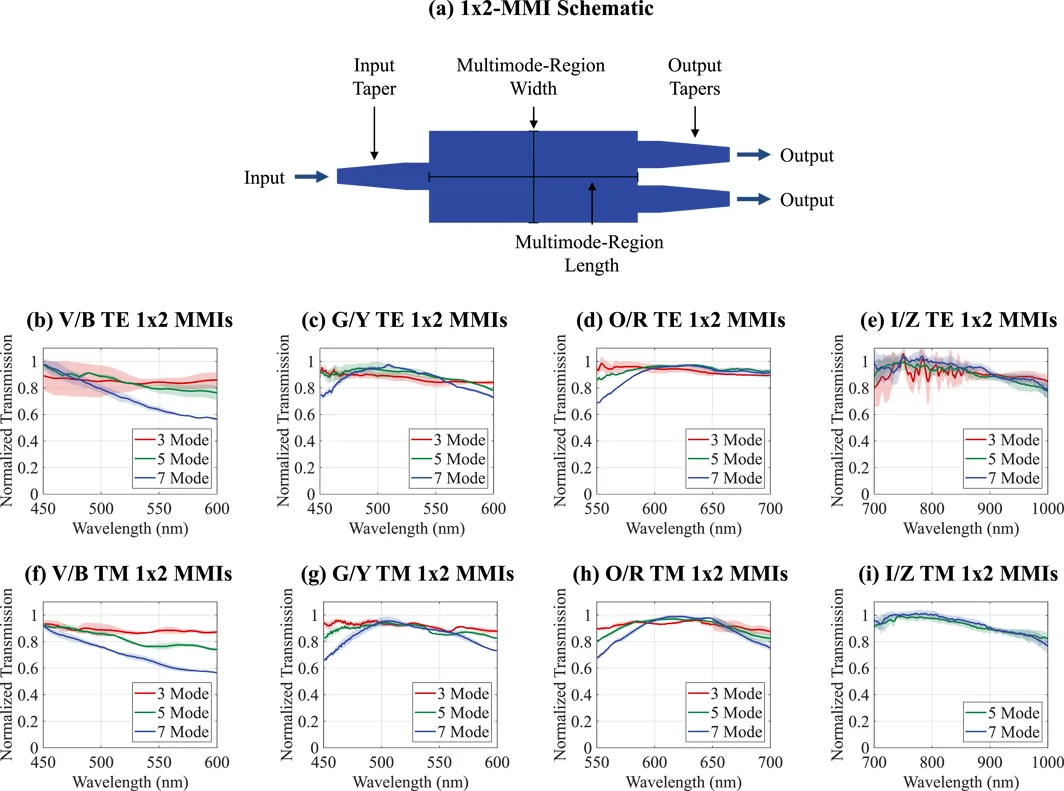
(a) Schematic of the 1‐x‐2 MMI splitter, with key design parameters labeled. Measured total normalized transmission of the 1‐x‐2 MMI splitters as a function of wavelength for designs targeting the V/B, G/Y, O/R, and I/Z bands and both (b–e) TE and (f–i) TM operation. Results are shown for 3 multimode‐region widths supporting 3 modes (red), 5 modes (green), and 7 modes (blue). Solid lines represent the weighted average across 3 reticles, and shaded regions represent the 1‐σ standard deviation.

### 6.2 2‐x‐2 MMI Splitters

The 2‐x‐2 MMI splitter is a general 3dB coupler with two input ports and two output ports, designed to evenly split and combine optical signals. In addition to serving as a power splitter, the 2‐x‐2 MMI is a key building block for MZI structures, where the splitting ratio accuracy, specifically the imbalance between the two output ports, directly determines the achievable extinction ratio of the interferometer. Even small deviations from an ideal 50/50 split can significantly degrade the extinction ratio, making low imbalance a critical performance metric for this device.

The 2‐x‐2 MMI consists of two input waveguides and two output waveguides connected to a multimode region, where general interference between the excited modes produces the desired power splitting [47]. Unlike the 1‐x‐2 MMI that relies on symmetric self‐imaging from a single‐centered input, the 2‐x‐2 MMI operates via general interference between modes excited by the offset input waveguides, resulting in complementary power transfer between the thru (input to same‐side output) and cross (input to opposite‐side output) ports. For the 2‐x‐2 MMI designs, we utilize the same multimode region widths, waveguide widths, and port offsets as those found for the 7‐mode 1‐x‐2 MMI devices described above, since these parameters are determined by the modal structure of the multimode region and are common to both device types [47]. We then reoptimize the length of the multimode region to achieve the desired 50/50 splitting ratio at the thru and cross output ports. As with the 1‐x‐2 MMIs, we design separate devices optimized for TE and TM inputs. The 2‐x‐2 MMI splitter parameters for designs targeting each of the V/B, G/Y, O/R, and I/Z bands and both TE and TM operation are summarized in Table 5.

**TABLE 5:** Parameters for the 2‐x‐2 MMI splitter designs targeting each of the V/B, G/Y, O/R, and I/Z bands and both TE and TM operation. The output‐port gap is the edge‐to‐edge separation between the two output tapers.

Parameter	V/B		G/Y		O/R		I/Z	
	TE	TM	TE	TM	TE	TM	TE	TM
Multimode‐region width	1.600 µm	1.850 µm	2.000 µm	2.450 µm	2.750 µm	3.550 µm	4.000 µm	5.000 µm
Multimode‐region length	21.614 µm	29.406 µm	28.592 µm	43.190 µm	43.306 µm	71.874 µm	73.008 µm	113.864 µm
Port gap	0.384 µm	0.497 µm	0.495 µm	0.694 µm	0.709 µm	1.062 µm	0.298 µm	0.767 µm
Taper width	0.456 µm	0.494 µm	0.555 µm	0.621 µm	0.729 µm	0.842 µm	1.494 µm	1.533 µm
Taper length	15 µm		15 µm		15 µm		25 µm	

Unlike the 1‐x‐2 MMI, where both output ports are symmetric by design and a single cascaded test structure is sufficient, the 2‐x‐2 MMI has distinct thru and cross output ports that may exhibit different transmission levels. To independently characterize the transmission to each port and quantify the splitting ratio asymmetry, we fabricate two separate cascaded test structures of four stages each for each designed device, analogous to the splitter‐tree structures described for the 1‐x‐2 MMIs. In the first test structure, the cascade is constructed such that the thru output of each stage feeds into the input of the next 2‐x‐2 MMI, whereas the cross output is routed to an edge coupler for measurement at each stage using the broadband setup described in Section 3. In the second test structure, the roles are reversed: the cross output feeds into the next stage and the thru output is routed to an edge coupler at each stage for measurement. Both test structures are measured for each design on three reticles on the wafer. By performing a linear fit of the measured power (in dB) versus the number of stages at each output edge coupler, we independently extract the per‐stage thru‐port and cross‐port transmission. The normalized splitting is then defined as the fraction of the total output power directed to each port, where an ideal 50/50 splitter would yield a normalized splitting of 0.5 for both the thru and cross ports.

The measured normalized splitting for both the thru port and the cross port of the 2‐x‐2 MMI splitters as a function of wavelength for designs targeting each of the V/B, G/Y, O/R, and I/Z bands and both TE and TM operation are shown in Figure 6, with the solid lines representing the weighted averages across three reticles and the shaded regions representing the 1‐σ standard deviations. Near the design wavelength for each band, the two port traces converge toward balanced splitting, with both ports receiving approximately equal power, indicating that the MMI length is well optimized at these wavelengths. Away from the design wavelength, the splitting ratio deviates from the 50/50, with the two ports diverging as the imaging condition shifts. The shaded standard‐deviation regions indicate good fabrication repeatability across most of the operating range, with somewhat more variability observed at the shorter‐wavelength ends of each band.

**FIGURE 6:**
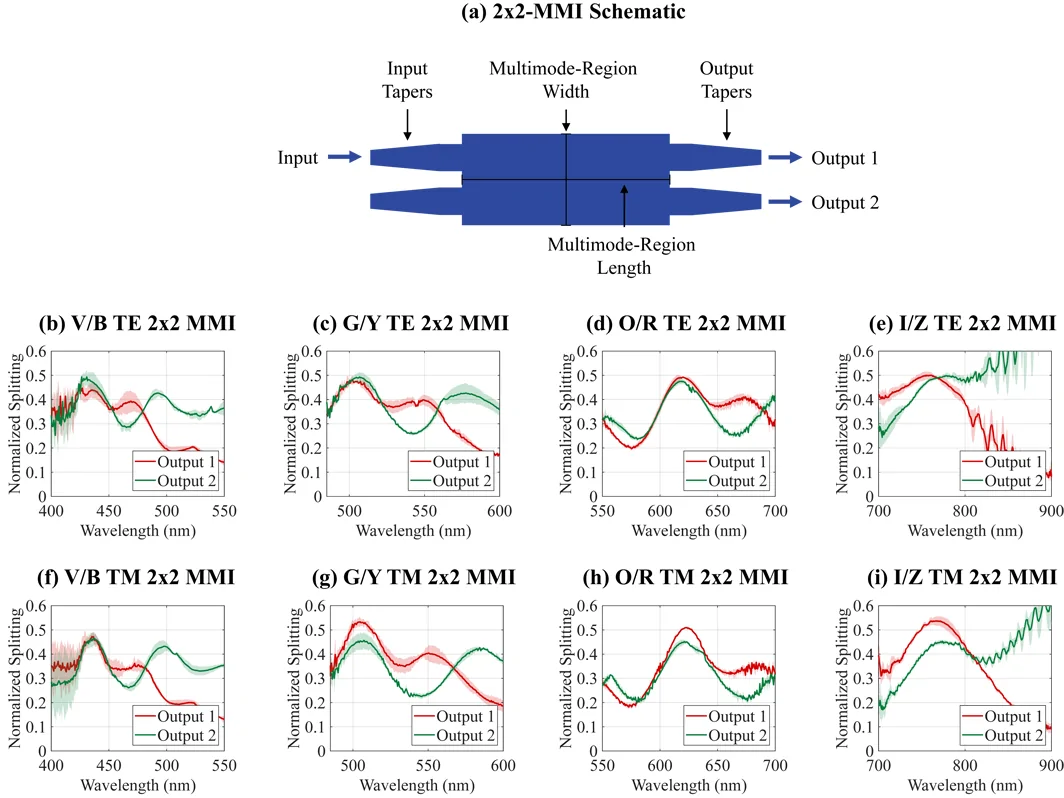
(a) Schematic of the 2‐x‐2 MMI splitter, with key design parameters labeled. Measured normalized power splitting between the two output ports of the 2‐x‐2 MMI splitters as a function of wavelength for designs targeting the V/B, G/Y, O/R, and I/Z bands and both (b–e) TE and (f–i) TM operation. Solid lines represent the weighted average across 3 reticles, and shaded regions represent the 1‐σ standard deviation.

### 6.3 Directional Couplers

Directional couplers provide an alternative approach to power splitting based on evanescent‐field coupling between two closely spaced waveguides. Unlike MMI splitters, which are designed for a fixed 50/50 splitting ratio, directional couplers can be designed for arbitrary splitting ratios. This flexibility makes directional couplers essential for applications requiring nonuniform power taps, such as power monitoring (using weak 99/1 or 90/10 taps), critical coupling to ring resonators, and the construction of lattice filters.

We utilize a multi‐stage directional‐coupler design to enable broadband performance across each wavelength band [48, 49]. In its simplest form, a directional coupler consists of two parallel waveguides brought into close proximity over a defined coupling length. The evanescent field of the guided mode in one waveguide overlaps with the adjacent waveguide, and the resulting coupling coefficient, which sets the splitting ratio, is determined by the gap between the waveguides and the coupling length. However, this standard single‐stage geometry produces a coupling ratio that varies strongly with wavelength, which is problematic for broadband applications. To achieve broadband coupling, the coupler is instead divided into two coupling stages separated by a delay region. The length difference between the two stages introduces a differential propagation phase between the coupled supermodes, which compensates for the wavelength dependence of the coupling, flattening the splitting ratio over a broad spectral range [48, 49]. By optimizing the gap, the lengths of the two coupling stages, and the length offset of the delay region, the splitting ratio can be made robust over a target design bandwidth. We optimize these parameters for directional couplers in 50/50, 90/10, and 99/1 splitting configurations for designs targeting each of the I/Z, O/R, and G/Y bands and both TE and TM operation. Because the evanescent field decays more rapidly at shorter wavelengths due to tighter mode confinement, achieving a given coupling strength requires proportionally smaller gaps for shorter wavelengths. As such, directional couplers for the V/B band have not yet been included in this release of the component library due to these tight gap requirements, but could be implemented in future iterations as fabrication processes are further optimized.

To characterize the directional couplers, we measure the transmission through both the thru and cross output ports using the broadband characterization setup described in Section 3. For each directional‐coupler design, we use test structures consisting of an input edge coupler, the directional coupler under test, and output edge couplers at both the thru and cross ports. The measured transmitted power at each port is normalized by the transmission through an adjacent reference waveguide on the same chip, yielding the normalized transmission to each port as a function of wavelength for each design.

The measured transmissions for the thru and cross ports of the directional couplers as a function of wavelength for the 50/50, 90/10, and 99/1 configurations for designs targeting each of the I/Z, O/R, and G/Y bands and both TE and TM operation are shown in Figure 7. Across all three bands and both polarizations, the directional couplers achieve their target splitting ratios near the design wavelength. The I/Z band exhibits the broadest and most stable performance, with all three configurations maintaining flat splitting ratios across the full 700‐to‐1000‐nm range for both polarizations. At shorter wavelengths, the splitting ratios become increasingly wavelength‐dependent, particularly for the 50/50 configurations as the smaller feature sizes make the coupling condition more sensitive to wavelength.

**FIGURE 7:**
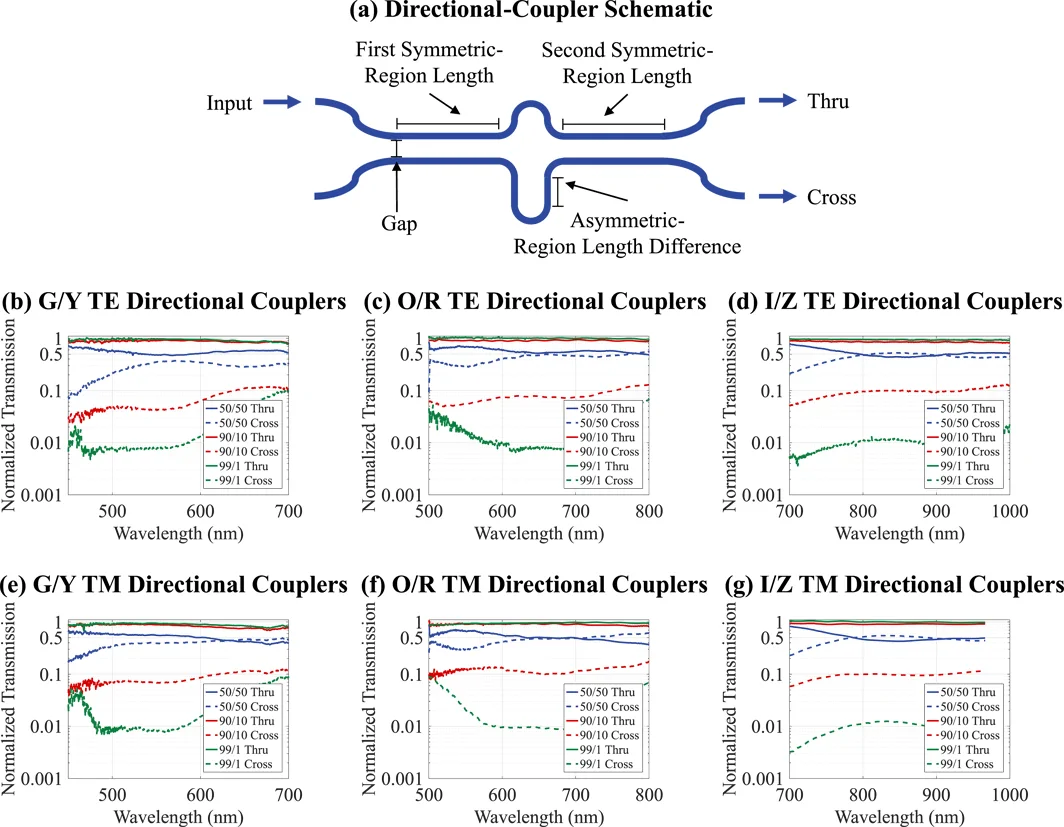
(a) Schematic of the directional coupler, with key design parameters labeled. Measured normalized transmission of the directional couplers as a function of wavelength for designs targeting the G/Y, O/R, and I/Z bands and both (b–d) TE and (e–g) TM operation. Thru‐port (solid lines) and cross‐port (dashed lines) transmissions are shown for the 50/50 (blue), 90/10 (red), and 99/1 (green) splitting configurations.

## 7 Crossings and Transitions: Waveguide Crossings and Inter‐Layer Escalators

Complex photonic integrated circuits require the ability to route waveguides across one another and to transition between different layers in a multi‐layer photonic stack. To enable this functionality in our platform, we design and characterize two key routing components: low‐crosstalk waveguide crossings and interlayer‐transition escalators for adiabatic transitions between the FN and SN layers.

### 7.1 Waveguide Crossings

Waveguide crossings enable two waveguides on the same layer to intersect with minimal insertion loss and low crosstalk between the crossing paths [50, 51]. Low‐crosstalk crossings are essential for dense photonic circuit layouts where waveguide routing inevitably requires signal paths to cross one another.

The waveguide crossings are designed in the FN layer, with the crossing geometry optimized independently for each spectral band to minimize scattering and crosstalk at the intersection point. We design two sets of these devices, one optimized for TE inputs and one for TM. The crossings use a compact multimode‐interference‐based design [50], in which the waveguide rapidly widens over a short taper into a multimode region before the intersection. This rapid expansion excites multiple guided modes within the widened region, and the length of this region is chosen such that the modes interfere constructively to produce a tightly focused field at the crossing point. This high spatial confinement at the intersection minimizes both the scattering loss and the power coupled into the perpendicular crossing waveguide. The widened‐region dimensions are optimized for each band to achieve the highest transmission through the crossing, therefore minimizing scattering and crosstalk. The waveguide‐crossing parameters for designs targeting each of the V/B, G/Y, O/R, and I/Z bands and both TE and TM operation are summarized in Table 6. Note that the simulated crosstalk into the perpendicular crossing waveguide is below 0.01% (approximately −43dB) per crossing for all of the designs.

**TABLE 6:** Parameters for the waveguide crossing designs targeting each of the V/B, G/Y, O/R, and I/Z bands and both TE and TM operation.

Parameter	V/B		G/Y		O/R		I/Z	
	TE	TM	TE	TM	TE	TM	TE	TM
Multimode‐region width	0.850 µm	0.850 µm	0.900 µm	0.850 µm	0.975 µm	1.150 µm	1.500 µm	1.800 µm
Multimode‐region length	4.684 µm	5.206 µm	4.562 µm	4.748 µm	4.514 µm	7.680 µm	8.274 µm	13.806 µm
Taper length	1.5 µm	1.5 µm	1.5 µm	1.5 µm	1.5 µm	2.0 µm	2.5 µm	4.0 µm

To characterize the waveguide crossings, for each waveguide‐crossing design, we use a suite of three test structures consisting of an input edge coupler leading to a section with varying numbers of crossings (50, 100, and 150 crossings) and, then, an output edge coupler. For each design, we measure the transmitted power as a function of number of crossings using the broadband setup described in Section 3 and perform a linear fit of the measured power (in dB) as a function of number of crossings to extract the insertion loss per crossing. For each crossing design, we measure one suite of test structures as described above on three reticles on the wafer.

The measured normalized transmissions of the waveguide crossings as a function of wavelength for designs targeting each of the V/B, G/Y, O/R, and I/Z bands and both TE and TM operation are shown in Figure 8, with the solid lines representing the weighted averages across three reticles and the shaded regions representing the 1‐σ standard deviations. Across all four bands, the waveguide crossings achieve high transmission, with normalized transmission values generally above 0.95 for both polarizations across most of the operating wavelength ranges. These results indicate that the devices introduce minimal excess loss per crossing, enabling dense waveguide routing with many sequential crossings without significant cumulative performance degradation.

**FIGURE 8:**
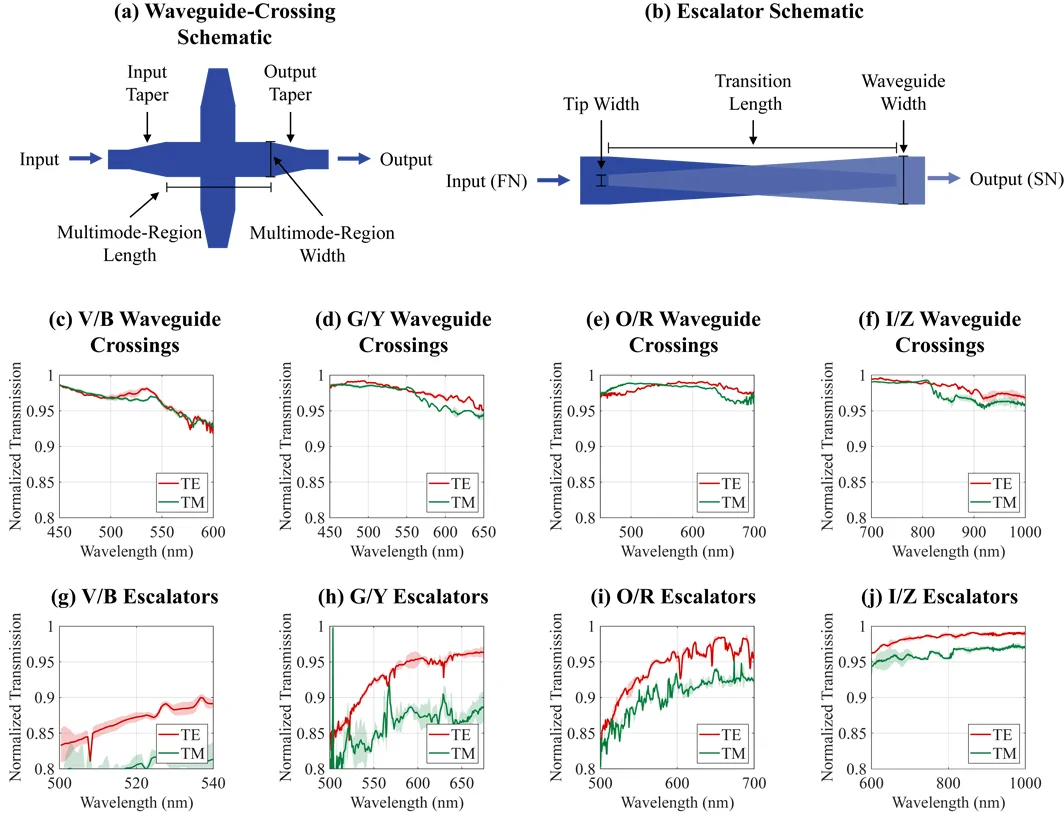
Schematic of the (a) waveguide crossing and (b) inter‐layer escalator, with key design parameters labeled. Measured normalized transmission of the waveguide crossings as a function of wavelength for designs targeting the (c) V/B, (d) G/Y, (e) O/R, and (f) I/Z bands and both TE (red) and TM (green) operation. Measured normalized transmission of the FN‐to‐SN interlayer‐transition escalators as a function of wavelength for designs targeting the (g) V/B, (h) G/Y, (i) O/R, and (j) I/Z bands and both TE (red) and TM (green) operation. Solid lines represent the weighted average across 3 reticles, and shaded regions represent the 1‐σ standard deviation.

### 7.2 Inter‐Layer Escalators

Interlayer‐transition escalators provide adiabatic optical transitions between the FN layer and the SN layer, enabling access to both waveguiding layers in the platform. This capability is valuable for several reasons: it enables bilayer waveguide routing, where signals can be routed on different layers to avoid crossings or increase routing density, it provides access to the thicker SN layer, which can support guided modes at longer wavelengths while keeping small bend radii, and it enables more complex device architectures that leverage both layers simultaneously such as polarization rotators.

The interlayer‐transition escalator operates by adiabatically transferring the optical mode from a waveguide on one layer to a waveguide on the other layer through evanescent coupling across the silicon‐dioxide gap between the FN and SN layers. The transition is achieved by tapering the width of the source waveguide down, while simultaneously tapering the width of the destination waveguide up, such that the effective index of the guided mode smoothly transitions from being confined in one layer to being confined in the other [52]. A single escalator geometry is designed to support both polarizations for each spectral band, with the taper tip widths set to 100 nm and the transition lengths set to 50 μm, optimized to ensure adiabatic transfer with minimal loss across the target wavelength range within a compact footprint. The escalator parameters for designs targeting each of the V/B, G/Y, O/R, and I/Z bands are summarized in Table 7.

**TABLE 7:** Parameters for the interlayer‐transition escalators for designs targeting each of the V/B, G/Y, O/R, and I/Z bands (a single design serves both the TE and TM polarizations within each band).

Parameter	V/B	G/Y	O/R	I/Z
FN waveguide width	250 nm	300 nm	400 nm	800 nm
SN waveguide width	250 nm	300 nm	325 nm	600 nm
Tip width	100 nm	100 nm	100 nm	100 nm
Transition length	50 µm	50 µm	50 µm	50 µm

To characterize the interlayer‐transition escalators, for each escalator design, we use a suite of three test structures containing varying numbers of pairs of escalators (10, 20, and 30 pairs, corresponding to 20, 40, and 60 escalators, with each pair consisting of one escalator that transitions the light from FN to SN and another that transitions back from SN to FN). For each design, we measure the transmitted power as a function of number of escalators using the broadband setup described in Section 3 and perform a linear fit of the measured power (in dB) as a function of number of escalators to extract the insertion loss per escalator. For each escalator design, we measure one suite of test structures as described above on three reticles on the wafer.

The measured normalized transmissions of the interlayer‐transition escalators as a function of wavelength for both the TE and TM input modes for designs targeting each of the V/B, G/Y, O/R, and I/Z bands are shown in Figure 8, with the solid lines representing the weighted averages across three reticles and the shaded regions representing the 1‐σ standard deviations. Since a single escalator geometry supports both polarizations within each band, the same design is used to obtain both the TE and TM results. The escalators achieve high transmission across all four bands and both polarizations, with normalized transmission values approaching unity at longer wavelengths in the O/R and I/Z bands. At shorter wavelengths, the transmission gradually decreases, reaching approximately 0.8 near 500 nm, which is consistent with the increased sensitivity of the adiabatic transition to wavelength as the mode confinement becomes tighter, and the minimum feature size determining the taper tip width becomes the limiting factor.

## 8 Polarization Management: Polarization Rotators and Polarization Splitters

Many visible‐wavelength applications involve unpolarized or mixed‐polarization optical inputs and outputs. For example, polarization‐gradient cooling schemes for trapped ions require precise delivery and manipulation of multiple polarization states simultaneously [23]. More broadly, polarization management is important for any application in which an unknown, random, or mixed polarization state is coupled to the photonic integrated circuit, including systems that collect unpolarized fluorescence or scattered Raman signals [11, 18]. To handle these signals on chip, polarization‐management devices that can separate and convert between the TE and TM modes are essential.

### 8.1 Polarization Rotators

Polarization rotators enable on‐chip mode conversion of an input TE mode to a TM mode, or vice versa [53, 54, 55, 56]. Ideally, when TE light is launched into the input, the rotator converts it entirely to TM at the output, and vice versa, with high extinction between the converted and unconverted polarization states. Low insertion loss for the desired polarization conversion and high extinction (low leakage of the unconverted polarization) are critical performance metrics, as any residual unconverted polarization degrades the polarization purity at the output. This functionality is important for a variety of visible‐wavelength polarization‐diverse architectures [22, 23, 55].

Our polarization‐rotator design is based on adiabatic mode evolution [53, 54, 55, 56] and consists of an input waveguide in the FN layer that is narrowed in the rotator section. An angled structure in the SN layer perturbs the waveguide mode and induces polarization rotation. Finally, a waveguide taper adiabatically transitions the rotated mode from the hybrid FN‐SN waveguide back into the FN waveguide at the rotator output. This design follows the approach of previous work on 780‐nm‐wavelength devices [56] and has been applied here to design polarization rotators targeting the G/Y, O/R, and I/Z bands.

To characterize the polarization rotators, we launch linearly polarized broadband light into the device using the broadband characterization setup described in Section 3. At the output, we use free‐space polarization optics to separately measure the power in the TE‐ and TM‐aligned polarization components and reconstruct the polarization rotation imparted by the device as a function of wavelength.

The measured normalized transmissions of the polarization rotators as a function of wavelength for designs targeting each of the G/Y, O/R, and I/Z bands are shown in Figure 9. The four traces in each panel represent the four possible input‐to‐output polarization combinations: TE‐to‐TE and TM‐to‐TM (which represent unwanted same‐polarization leakage through the rotator, ideally minimized), and TE‐to‐TM and TM‐to‐TE (which represent the desired polarization rotation, ideally maximized). Across all 3 bands, the rotators achieve effective polarization conversion, with the desired cross‐polarization transmission (TE‐TM and TM‐TE) approximately 15–20 dB higher than the unwanted same‐polarization transmission (TE‐TE and TM‐TM) near the design wavelengths, indicating good extinction between the rotated and unrotated outputs.

**FIGURE 9:**
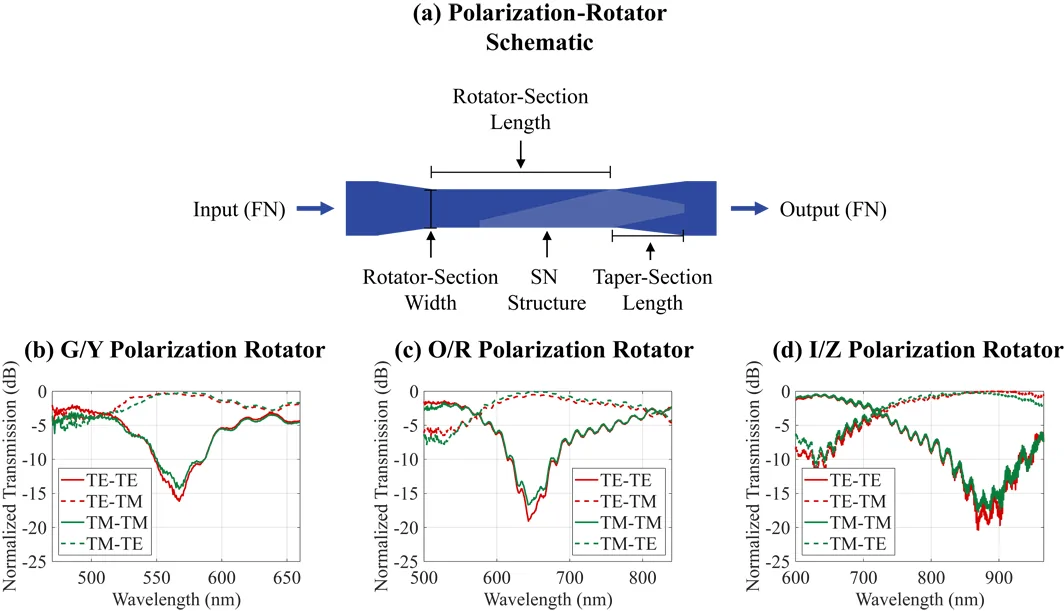
(a) Schematic of the polarization rotator, with key design parameters labeled. Measured normalized transmission (dB) of the polarization rotators as a function of wavelength for designs targeting the (b) G/Y, (c) O/R, and (d) I/Z bands. The 4 traces represent the input‐to‐output polarization combinations: TE‐to‐TE (red solid), TE‐to‐TM (red dashed), TM‐to‐TM (green solid), and TM‐to‐TE (green dashed).

### 8.2 Polarization Splitters

Polarization splitters separate an input containing both TE and TM components into different output waveguides. Ideally, when TE light is launched into the input port, it is directed entirely to the output cross port, and, when TM light is launched into the input port, it is directed entirely to the output thru port, with high extinction between the desired and undesired outputs at each port [55]. Low insertion loss for the desired polarization and high extinction (low crosstalk from the unwanted polarization) are critical performance metrics as any leaked power from the unwanted polarization into a given port degrades the polarization purity of the output.

The polarization splitters are based on directional couplers in which the gap and coupling length are optimized to exploit the polarization‐dependent coupling strength between two closely‐spaced waveguides [55]. Because the TE and TM modes have different effective indices and evanescent‐field profiles, the coupling coefficient differs for each polarization for a given gap, allowing the coupler geometry to be designed such that one polarization is routed to the cross port whereas the other remains in the thru port. We design polarization splitters targeting 532 nm in the G/Y band, 633 nm in the O/R band, and 785 nm in the I/Z band.

To characterize the polarization splitters, we measure the transmission through both the thru and cross output ports for both TE and TM input polarizations using the broadband characterization setup described in Section 3. At the output, we use free‐space polarization optics to separately measure the TE‐ and TM‐aligned polarization components at each port. The measured transmitted power at each port is normalized by the transmission through an adjacent reference waveguide on the same chip, yielding the normalized transmission for each polarization to each port as a function of wavelength for each design.

The measured normalized transmissions of the polarization splitters as a function of wavelength for designs targeting each of the G/Y, O/R, and I/Z bands are shown in Figure 10. The 4 traces in each panel represent the TE and TM transmission to both the thru and cross ports. An ideal polarization splitter would direct one polarization entirely to the thru port and the other entirely to the cross port, with high extinction between them. Near the design wavelengths, the splitters achieve extinction ratios of approximately 20 dB or greater between the desired and undesired polarization outputs, indicating high polarization purity.

**FIGURE 10:**
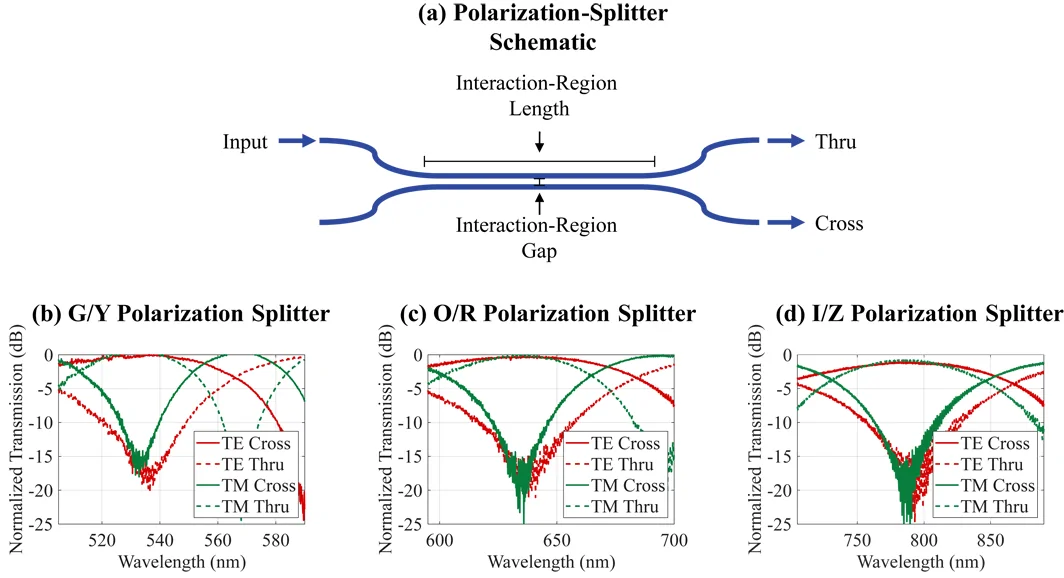
(a) Schematic of the polarization splitter, with key design parameters labeled. Measured normalized transmission (dB) of the polarization splitters as a function of wavelength for designs targeting the (b) G/Y, (c) O/R, and (d) I/Z bands. The 4 traces represent the TE‐input‐to‐cross‐port (red solid), TE‐input‐to‐thru‐port (red dashed), TM‐input‐to‐cross‐port (green solid), and TM‐input‐to‐thru‐port (green dashed) transmission.

## 9 Sensing Compatibility: Sensing‐Trench Propagation and Transitions

One major differentiator of this platform compared to other visible‐wavelength silicon‐photonics platforms is the inclusion of a sensing trench, which removes the silicon‐dioxide cladding above the FN waveguide in designated regions and allows the evanescent field of the guided optical mode to interact directly with an external medium. This capability is critical for on‐chip sensing applications, such as WERS, biosensing, and chemical detection, where the sensitivity of the sensor is directly determined by the strength of the interaction between the guided light and the analyte. We characterize two key metrics for the sensing trench: the transition loss incurred when the waveguide enters and exits the sensing region and the propagation loss within the sensing trench itself.

When a waveguide transitions from the fully oxide‐clad region into the sensing trench, the optical mode experiences an abrupt change in the cladding refractive index, from silicon dioxide to air (or the analyte medium). This cladding discontinuity causes a mismatch between the mode profiles in the two regions, leading to scattering and reflection at the transition interface. In addition, other factors can contribute to loss at this interface, such as process‐dependent etching of the waveguide core or the presence of residual SN material. Minimizing this transition loss is important for sensing system performance as each sensing region requires two transitions (one at the entrance and one at the exit), and the cumulative transition loss reduces the optical power available for interaction with the analyte as well as reducing the signal from the analyte.

Within the sensing trench, the waveguide is clad by air (or the analyte‐sensing medium) rather than silicon dioxide, resulting in a different propagation loss compared to the fully clad waveguide regions characterized in Section 4. The change in cladding modifies the mode profile and confinement, and the exposed waveguide surface may introduce additional scattering loss due to surface roughness or contamination in the trench. Characterizing the propagation loss within the sensing trench is essential for designing sensing systems with known loss budgets as it determines how long the sensing interaction region can be before the guided power is excessively attenuated.

To characterize both the sensing trench transition loss and the propagation loss within the sensing trench, we use a set of test structures that each contain a different number of trench segments but maintain the same total sensing trench length. The test structures consist of waveguides that pass through 1, 2, 4, and 8 trench segments, where each configuration is designed such that the combined length of all trench segments is held constant. This means that, as the number of trench segments increases, the individual segments become shorter but the total length of waveguide under the sensing trench remains the same. Consequently, the total propagation loss within the sensing trench is constant across all test structures, and the only quantity that varies is the number of trench interface pairs (entrance and exit transitions). We first normalize the transmitted power through each test structure to the power measured through a reference straight waveguide of the same total length but without any sensing trench, removing the contribution of the fully clad waveguide propagation loss and coupling losses. We then perform a linear regression of the normalized loss (in dB) versus the number of trench interface pairs at each wavelength. The slope of this fit yields the insertion loss per sensing‐trench interface, which quantifies the loss incurred each time the waveguide transitions into and out of the sensing trench. Additionally, the *y* intercept of the fit yields the baseline loss associated with propagating through the total sensing trench length, which we divide by the total trench length to obtain the sensing trench propagation loss. These measurements are performed using the broadband characterization setup described in Section 3 for three FN waveguide widths (400 nm, 500 nm, and 800 nm) and both TE and TM input polarization. Note that all sensing trench measurements are performed with air as the top cladding. For each waveguide width, we measure one suite of test structures as described above on three reticles on the wafer.

The measured insertion loss per trench transition as well as trench propagation loss as a function of wavelength for the 400‐nm, 500‐nm, and 800‐nm‐wide waveguides are shown in Figure 11, with the solid lines representing the weighted averages across three reticles and the shaded regions representing the 1‐σ standard deviations. The TM data is not shown for the 800‐nm‐wide waveguide as the fundamental TM mode is not supported at the longer wavelengths of the I/Z band due to the waveguide thickness being too thin relative to the wavelength to confine the TM mode. Additionally, the TM trench‐transition and propagation‐loss data exhibit periodic spectral fringes, which are likely caused by parasitic Fabry–Perot reflections at the trench interfaces within the test structures.

**FIGURE 11:**
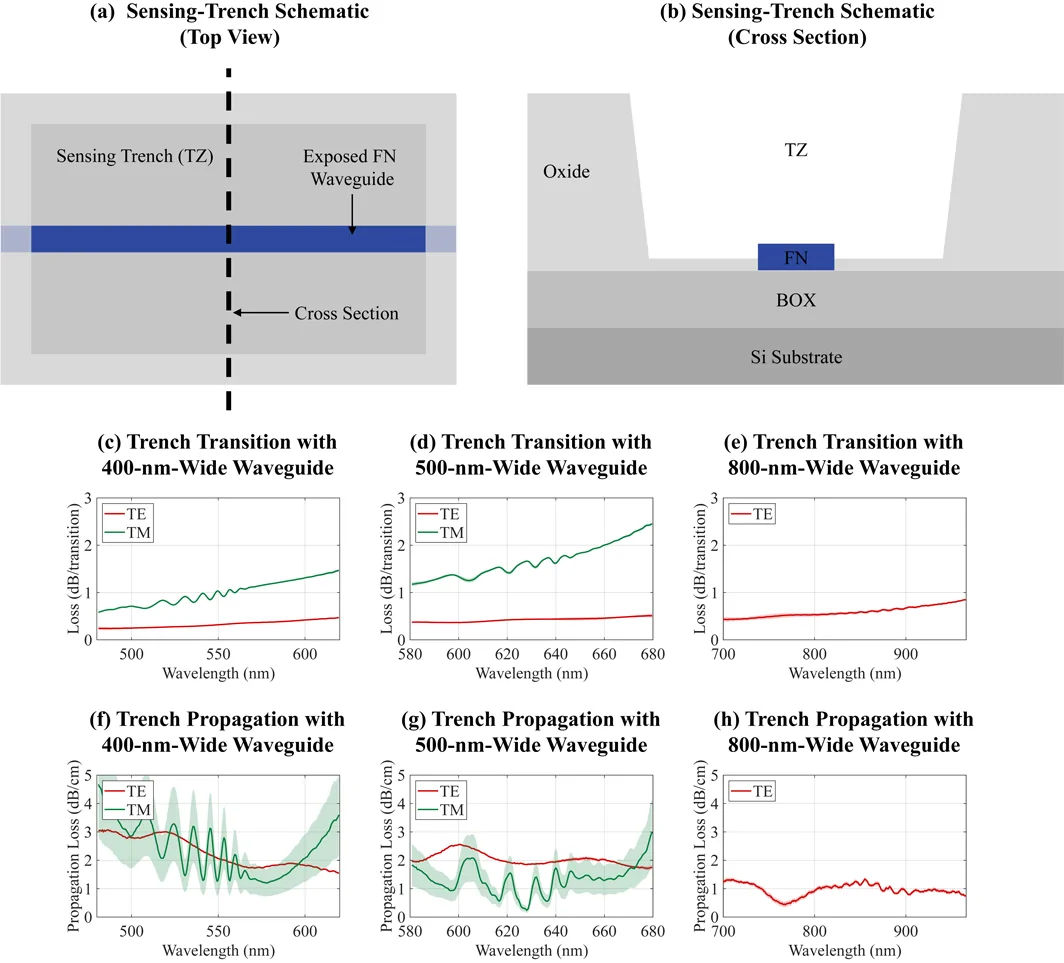
(a) Top‐view and (b) cross‐sectional schematic of the sensing trench, with key design parameters labeled. Measured insertion loss (dB/transition) for entering or exiting the air‐clad sensing trench region as a function of wavelength for (c) 400‐nm‐wide, (d) 500‐nm‐wide, and (e) 800‐nm‐wide waveguides for both TE (red) and TM (green) operation. Measured propagation loss (dB/cm) within the air‐clad sensing trench as a function of wavelength for the (f) 400‐nm‐wide, (g) 500‐nm‐wide, and (h) 800‐nm‐wide waveguides for both TE (red) and TM (green) operation. Solid lines represent the weighted average across 3 reticles, and shaded regions represent the 1‐σ standard deviation.

## 10 Active Modulation: Thermo‐Optic Phase Shifters

Active modulation is essential for reconfigurable photonic integrated circuits, enabling applications such as programmable interferometers, tunable filters, and non‐mechanical beam steering [26, 36, 37, 57, 58]. To enable this functionality in our platform, we design and characterize thermo‐optic phase shifters that leverage the metallization stack described in Section 2 to provide electrically controlled phase tuning of the guided optical mode.

The thermo‐optic phase shifters operate by resistively heating the silicon‐dioxide cladding region surrounding the waveguide, which locally modifies the refractive index of the cladding and core materials through the thermo‐optic effect, inducing a phase shift on the guided mode [36, 37]. In our platform, the heater element consists of a resistor patterned above the waveguide as described in Section 2. Electrical current is delivered to the heater through the metal via layer, which provides electrical contact up to the top metal layer that defines both the electrical routing traces and the top‐level electrical pads for interfacing with electrical probes or wire bonding. Although our platform does also support undercut heater geometries for improved thermal efficiency [37], we present the nonundercut designs here as the standard offering for the component library as they provide greater mechanical robustness and fabrication simplicity and are sufficient for many applications. We design thermo‐optic phase shifters for all five waveguide widths in the component library (250 nm, 300 nm, 400 nm, 500 nm, and 800 nm), spanning the full spectral range from the V/B to the I/Z band. The heater parameters for designs targeting each of the V/B, G/Y, O/R, and I/Z bands are summarized in Table 8.

**TABLE 8:** Parameters for the thermo‐optic phase shifter for designs targeting each of the V/B, G/Y, O/R, and I/Z bands (a single design serves both the TE and TM polarizations within each band).

Parameter	V/B	G/Y	O/R	I/Z
Heater width	3 µm	3 µm	3 µm	3 µm
Heater length	1000 µm	1000 µm	1000 µm	1000 µm
Waveguide width	250 nm	300 nm	400 nm	800 nm

To characterize the thermo‐optic phase shifters, we perform both electrical and optical measurements. First, we perform current–voltage (I–V) characterization of the heaters to verify the resistance and linear ohmic behavior of the heater elements. The heaters exhibit linear ohmic behavior with resistances of approximately 4.5 kΩ across all designs. Next, to extract the optical phase‐shifting efficiency, we use the broadband characterization setup described in Section 3 to measure the optical output of a Mach–Zehnder interferometer (MZI) test structure in which the thermo‐optic phase shifter is placed on one arm of the MZI. We land electrical probes on the pads connected to the phase shifter and sweep the applied electrical power while recording the MZI output power, observing the characteristic sinusoidal interference fringe pattern as the differential phase between the two arms is swept. The electrical power required to shift the MZI output from a maximum to an adjacent minimum (or vice versa) corresponds to the power required for a π phase shift, denoted P_π_, which is the standard figure of merit for thermo‐optic phase‐shifter efficiency, with lower P_π_ values indicating more efficient phase shifting. Finally, we characterize the temporal response of the phase shifters by applying a square‐wave electrical signal to the heater and recording the optical output of the MZI using a 633‐nm laser and a photodetector (instead of using the broadband source and the spectrometer used for the spectral measurements).

The measured P_π_ of the thermo‐optic phase shifters as a function of wavelength for both TE and TM input polarizations across all five waveguide widths are shown in Figure 12, with the solid lines representing the weighted averages across three reticles and the shaded regions representing the 1‐σ standard deviations. Across all waveguide widths, P_π_ increases with wavelength. This is primarily because a given refractive‐index change produces a smaller phase shift at longer wavelengths, requiring more heating to achieve a π phase shift. This trend is further reinforced by the weaker confinement of longer‐wavelength modes in the waveguide core, where the thermo‐optic effect is strongest. The TE mode consistently requires less power than the TM mode to achieve a π phase shift across all widths and wavelengths, which is also attributed to the stronger confinement of the TE mode within the waveguide core. Across all these measurements, the phase shifters were driven at electrical powers of up to 300 mW without failure. The measured 3‐dB modulation bandwidth of the phase shifters is approximately 6.5 kHz across all designs, consistent with typical oxide‐clad thermo‐optic devices. As noted above, these P_π_ values are obtained using standard nonundercut heater geometries; undercut designs, which this platform also supports, have demonstrated up to a 27x reduction in P_π_ [37].

**FIGURE 12:**
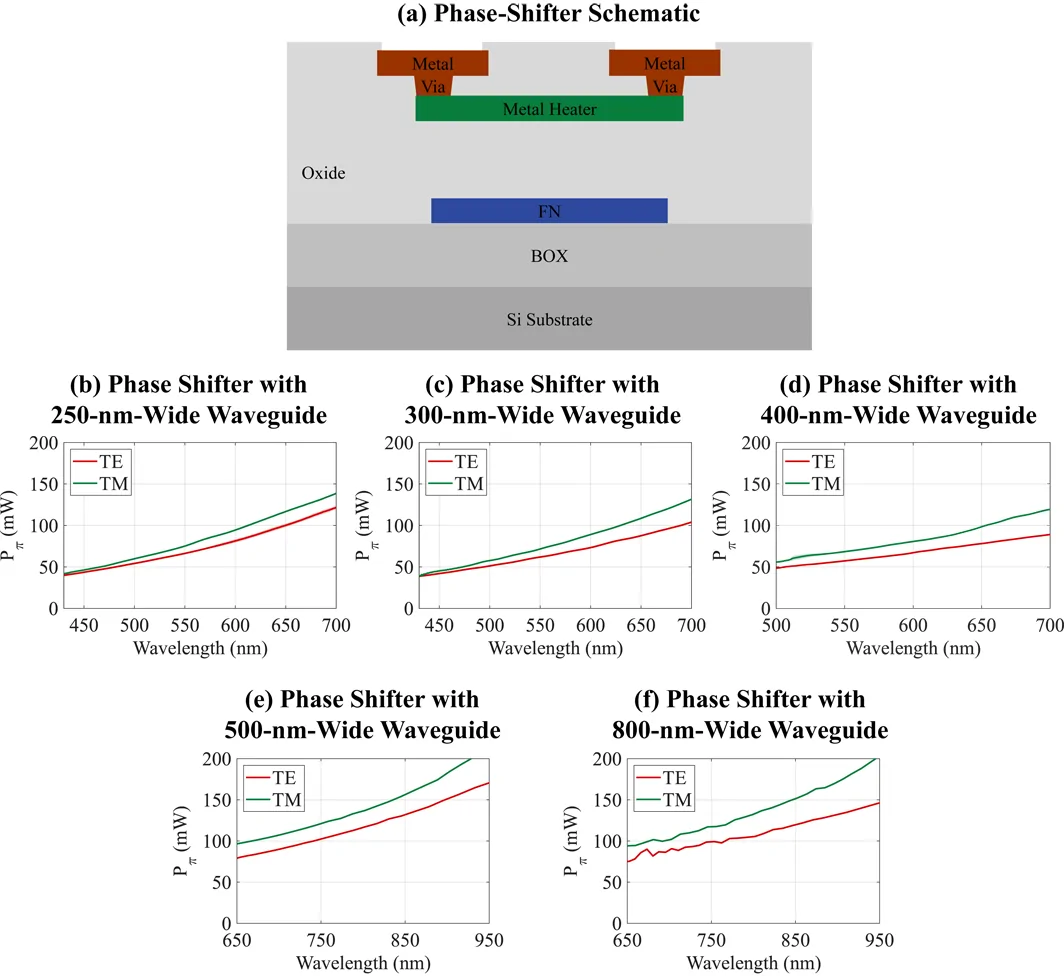
(a) Cross‐sectional schematic of the thermo‐optic phase shifter, with key design parameters labeled. Measured power required for a π phase shift (P_π_) for the thermo‐optic phase shifters as a function of wavelength for (b) 250‐nm‐wide, (c) 300‐nm‐wide, (d) 400‐nm‐wide, (e) 500‐nm‐wide, and (f) 800‐nm‐wide waveguides for both TE (red) and TM (green) operation. Solid lines represent the weighted average across 3 reticles, and shaded regions represent the 1‐σ standard deviation.

## 11 Conclusion

In this work, we presented the design, fabrication, and broadband experimental characterization of a comprehensive visible‐to‐near‐infrared‐spanning component library for the AIM‐Photonics 300‐mm‐wafer silicon‐nitride‐based platform, which features two silicon‐nitride waveguiding layers, a sensing trench, thermo‐optic phase shifters for active modulation, and a dicing trench for smooth fiber‐to‐chip edge coupling. We designed and characterized components for both the fundamental TE and TM modes for wavelengths spanning from 450 to 1000 nm and covering the V/B, G/Y, O/R, and I/Z bands. To enable this broadband characterization, we utilized a spectrum‐spanning measurement setup and methodology capable of testing devices across the full 450‐to‐1000‐nm wavelength range. The component library includes passive waveguides with propagation‐loss characterization across multiple waveguide widths (with TE single‐mode propagation losses as low as 4.5 dB/cm in the V/B band and decreasing to 0.1 dB/cm in the I/Z band), broadband fiber‐to‐chip edge couplers (with insertion losses as low as 4.3 dB/facet in the V/B band and decreasing to 1.9 dB/facet in the I/Z band), fiber‐to‐chip grating couplers (with insertion losses as low as 5.4 dB/coupler), 1‐x‐2 and 2‐x‐2 MMI splitters, directional couplers with 50/50, 90/10, and 99/1 splitting ratios, low‐crosstalk waveguide crossings, interlayer‐transition escalators for adiabatic transitions between the two silicon‐nitride layers, polarization rotators, polarization splitters, sensing‐trench transitions and propagation structures, and thermo‐optic phase shifters. This work provides the silicon‐photonics community with a foundry‐based pre‐characterized library of photonic building blocks for visible‐to‐near‐infrared applications.

In the future, we plan to extend this component library and platform in several directions. First, we aim to integrate on‐chip optical sources, such as heterogeneously‐integrated lasers or rare‐earth‐doped gain materials [59, 60, 61, 62], to enable fully self‐contained photonic systems that do not require external laser coupling. Second, we plan to integrate on‐chip photodetectors to enable closed‐loop sensing and signal processing directly on the photonic chip [63]. Third, we will work to extend the entire component library to shorter wavelengths in the V/B band, where several components, such as directional couplers and polarization management devices, have not yet been included due to the tighter fabrication tolerances required at these wavelengths. Fourth, we will explore extending the platform toward ultraviolet wavelengths, which would open up additional application areas in spectroscopy and quantum sensing with ion species that have transitions below 450 nm. Together, these efforts will continue to advance the capabilities of visible‐wavelength silicon photonics and broaden the range of applications that can be addressed using this foundry platform.

This work addresses a critical gap in the visible‐wavelength silicon‐photonics ecosystem. Although infrared silicon‐photonics platforms have benefited from mature foundry processes with comprehensive PDKs and component libraries for over a decade, visible‐wavelength platforms have lacked equivalent resources, limiting the ability of designers to rapidly prototype and deploy photonic integrated circuits for the many emerging applications that require visible‐wavelength operation. By providing a complete component library in a standard 300‐mm foundry platform with sub‐100‐nm lithographic resolution, this work lowers the barrier to entry for designing visible‐wavelength photonic integrated circuits and enables the broader photonics community to leverage the same foundry infrastructure that has driven the success of infrared silicon photonics.

## Author Contributions

**Andres Garcia Coleto:** data curation, methodology, formal analysis, writing – original draft, writing – review and editing. **Henry Crawford‐Eng:** data curation, formal analysis, writing – original draft, methodology, writing – review and editing. **Theodore J. Dahl:** data curation, formal analysis, methodology, writing – review and editing. **Mariel Shapiro:** data curation, formal analysis, methodology. **Maria Schnuck:** methodology, formal analysis, data curation. **Kyle J. Walsh:** data curation, formal analysis, methodology, writing – review and editing. **Steven T. Lipkowitz:** data curation, formal analysis, methodology, writing – review and editing. **Marcel W. Pruessner:** data curation, formal analysis, methodology, writing – review and editing. **Nathan F. Tyndall:** data curation, formal analysis, methodology, writing – review and editing. **Jacob N. Bouchard:** methodology, data curation, formal analysis, writing – review and editing. **Alin Antohe:** methodology, data curation, formal analysis. **Soumen Kar:** data curation, formal analysis, methodology. **Patrick J. O'Mullan:** methodology, data curation, formal analysis, writing – review and editing. **Cooper Hurley:** data curation, formal analysis, methodology, writing – review and editing. **Peter Hedlesky:** data curation, formal analysis, methodology, writing – review and editing. **Vijay S. S. Sundaram:** methodology, data curation, formal analysis. **Stefan Preble:** supervision. **Kevin McComber:** supervision, writing – review and editing. **James Eakin:** supervision, writing – review and editing. **Doug T. Petkie:** supervision, writing – review and editing. **Lewis G. Carpenter:** writing – review and editing, supervision. **Todd H. Stievater:** supervision, writing – review and editing. **Jelena Notaros:** writing – review and editing, writing – original draft, supervision.

## Conflicts of Interest

The authors declare no conflicts of interest.

## Data Availability

Data are available upon reasonable request to the corresponding author.
